# Integrin-mediated mTOR signaling drives TGF-**β** overactivity and myxomatous mitral valve degeneration in hypomorphic fibrillin-1 mice

**DOI:** 10.1172/JCI183558

**Published:** 2025-05-20

**Authors:** Fu Gao, Qixin Chen, Makoto Mori, Sufang Li, Giovanni Ferrari, Markus Krane, Rong Fan, George Tellides, Yang Liu, Arnar Geirsson

**Affiliations:** 1 Division of Cardiac Surgery, Department of Surgery, Yale School of Medicine, New Haven, Connecticut, USA.; 2 Department of Biomedical Engineering, Yale University, New Haven, Connecticut, USA.; 3 Department of Pathology, Yale School of Medicine, New Haven, Connecticut, USA.; 4 Department of Surgery and; 5 Columbia Surgical Cardiovascular Research Institute, Columbia University, New York, New York, USA.; 6 Department of Cardiovascular Surgery, Institute Insure, German Heart Center Munich, School of Medicine and Health, Technical University of Munich, Munich, Germany.; 7 DZHK (German Center for Cardiovascular Research), Partner Site Munich Heart Alliance, Munich, Germany.; 8 Yale Stem Cell Center,; 9 Yale Cancer Center,; 10 Yale Center for Research on Aging (Y-Age),; 11 Human and Translational Immunology Program, and; 12 Program in Vascular Biology and Therapeutics, Yale School of Medicine, New Haven, Connecticut, USA.; 13 Veterans Affairs Connecticut Health Care System, West Haven, Connecticut, USA.; 14 Department of Neurology, Yale School of Medicine, New Haven, Connecticut, USA.

**Keywords:** Cardiology, Cell biology, Cardiovascular disease, Extracellular matrix, Signal transduction

## Abstract

Mitral valve prolapse is often benign, but progression to mitral regurgitation may require invasive intervention and there is no specific medical therapy. An association of mitral valve prolapse with Marfan syndrome resulting from pathogenic *FBN1* variants supports the use of hypomorphic fibrillin-1 mgR mice to investigate mechanisms and therapy for mitral valve disease. mgR mice developed severe myxomatous mitral valve degeneration with mitral regurgitation by 12 weeks of age. Persistent activation of TGF-β and mTOR signaling along with macrophage recruitment preceded histological changes at 4 weeks of age. Short-term mTOR inhibition with rapamycin from 4 to 5 weeks of age prevented TGF-β overactivity and leukocytic infiltrates, while long-term inhibition of mTOR or TGF-β signaling from 4 to 12 weeks of age rescued mitral valve leaflet degeneration. Transcriptomic analysis identified integrins as key receptors in signaling interactions, and serologic neutralization of integrin signaling or a chimeric integrin receptor altering signaling prevented mTOR activation. We confirmed increased mTOR signaling and a conserved transcriptome signature in human specimens of sporadic mitral valve prolapse. Thus, mTOR activation from abnormal integrin-dependent cell–matrix interactions drives TGF-β overactivity and myxomatous mitral valve degeneration, and mTOR inhibition may prevent disease progression of mitral valve prolapse.

## Abstract 2

Myxomatous mitral valve degeneration and mTOR activation, evident in hypomorphic fibrillin-1 mice and human specimens can be modulated by rapamycin in-vivo.

## Graphical abstract

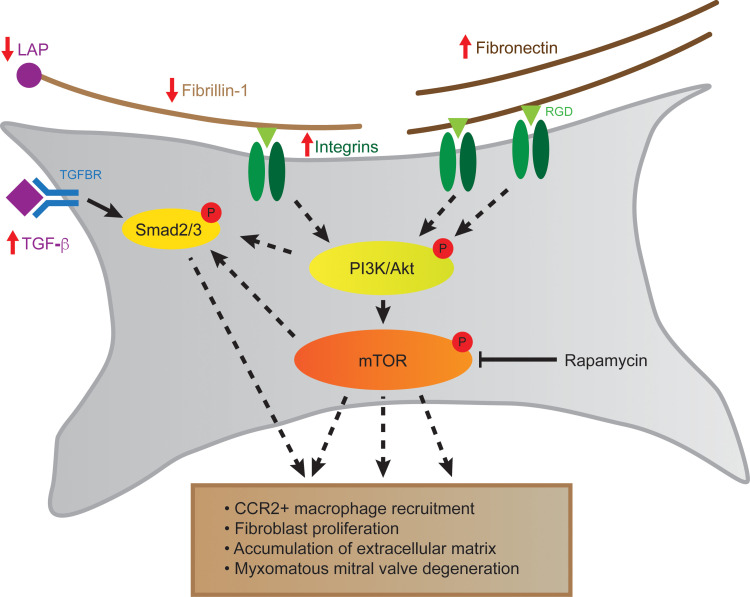

## Introduction

Mitral valve prolapse (MVP) is the most common acquired cardiac valvular abnormality in industrialized nations with a prevalence of 0.6%–2.4% (1). Up to a quarter of individuals with MVP develop pathological mitral regurgitation (MR) that requires surgical or transcatheter intervention within 15 years (2). There is no medical therapy to prevent or slow the progression of MVP. Well-characterized histological changes associated with MVP include myxomatous degeneration with leaflet thickening and lengthening, accumulation of an extracellular matrix (ECM) rich in glycosaminoglycans (GAGs), and disruption of normal ECM architecture with collagen dissolution (3, 4). Cellular components of myxomatous disease include activated fibroblast-like valvular interstitial cells that mediate ECM remodeling and recruited macrophages that are primarily CCR2 positive and promote inflammation (5–7). MVP, in addition to other cardiovascular abnormalities such as aortic dilatation, also occurs in patients with Marfan syndrome (MFS) resulting from pathogenic *FBN1* variants (8). *Fbn1^C1041G/+^* heterozygous mice, which contain a similar variant to the human FBN1 Cys1039Tyr pathogenic variant representing a common mechanism of disease for MFS with substitution of a cysteine in the calcium-binding EGF-like domain, develop typical skeletal and cardiovascular abnormalities though modest in severity and without clinical endpoints of lethality (9). Although the exact mechanism is not fully defined, the current understanding of MVP and aortic aneurysm formation in MFS is that altered matrix sequestration of latent TGF-β increases bioavailability of TGF-β, and increased signaling drives disease pathogenesis rather than deficient structural integrity of tissue from abnormal fibrillin-1 microfibrils (10). This hypothesis is supported by phenotypic rescue of cardiovascular defects, including early myxomatous mitral valve degeneration, in *Fbn1^C1041G/C1041G^* mice by TGF-β neutralization (11). Other signaling abnormalities implicated in mitral valve disease include Wnt/β-catenin activation through Axin2 and serotonin transporter deficiency in mouse models and clinical specimens (12–14). However, the mechanism(s) involved in the pathogenesis of sporadic MVP in humans remains elusive.

We used hypomorphic fibrillin-1 (*Fbn1^mgR/mgR^*, abbreviated as mgR) mice that develop severe myxomatous mitral valve degeneration to investigate signaling pathway perturbations before and after tissue remodeling, including mTOR signaling. A key role for mTOR activity was established by complete rescue of the mitral valve disease phenotype by rapamycin treatment of mgR mice, in which the pathological process was dependent on both integrin and TGF-β signaling. Increased β_1_ integrin expression and mTOR overactivity was found in human tissue specimens of MVP, and single-cell RNA-Seq analysis indicated conserved transcriptional responses between murine models and humans. Identification of mTOR as a therapeutic target for preventing the progression of myxomatous mitral valve disease in a mouse model and evidence of mTOR activation in human specimens of sporadic MVP hold important translational implication given the availability of several mTOR inhibitors approved for clinical use.

## Results

### Fibrillin-1 deficiency in mgR mice results in severe myxomatous mitral valve degeneration associated with activation of mTOR and TGF-β signaling and recruitment of macrophages at 12 weeks of age.

**Figure 1:**
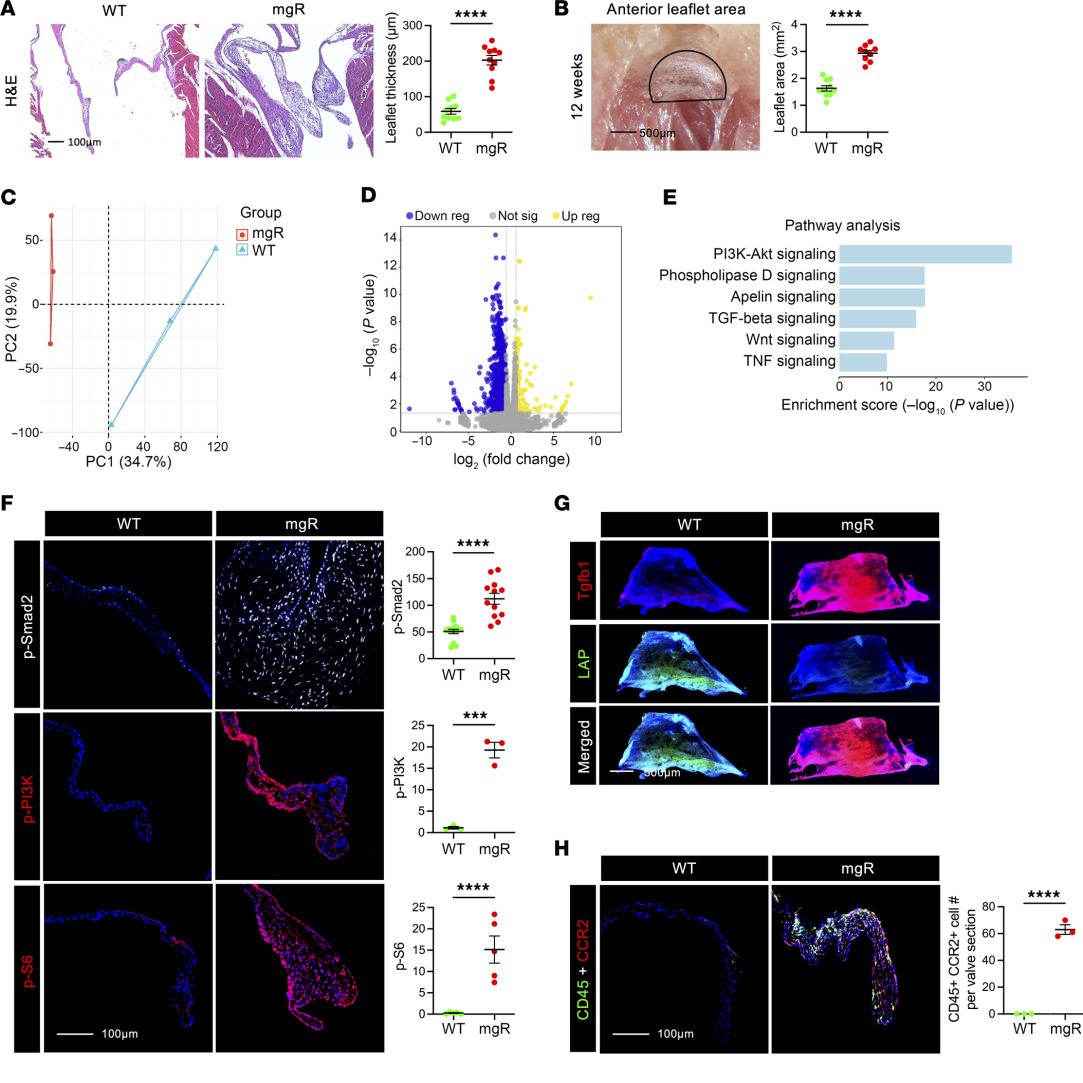
Fibrillin-1 deficiency associated with severe myxomatous mitral valve disease as well as TGF-β and mTOR signaling activation in 12-week-old mgR mice. (**A**) H&E staining of long-axis sections of mitral valve leaflets in 12-week-old WT versus mgR mice and measurement of maximal leaflet thickness (*n* = 10). Scale bar: 100 μm. (**B**) Morphometric analysis of anterior mitral valve leaflet area (outlined by a black semicircle) (*n* = 9–10). Scale bar: 500 μm. Bulk RNA-Seq data from mitral valves analyzed by (**C**) principal component analysis, (**D**) volcano plot of DEGs, and (**E**) GO pathway analysis based on input genes with adjusted *P* < 0.05 (*n* = 3). (**F**) Representative IF and mean fluorescence density measurements, in AU, of mitral valve leaflets for p-Smad2 (*n* = 12), p-PI3K (*n* = 3), and p-S6 (*n* = 5). Scale bar: 100 μm. (**G**) Representative whole-mount IF of anterior mitral valve leaflet for TGF-β1 (Tgfb1) and LAP. Scale bar: 500 μm. (**H**) Representative IF of CD45 and CCR2 staining and number of CD45^+^/CCR2^+^ cells per valve section (*n* = 3). Scale bar: 100 μm. Data are represented as individual values with mean ± SEM; ***P < 0.001, ****P < 0.0001 by unpaired *t* test.

To investigate whether fibrillin-1 insufficiency causes MVP, we used homozygous mgR mice that express ~30% fibrillin-1 and are viable during postnatal development (15). *Fbn1^+/+^* (WT) littermates were used as controls for each experimental condition to account for possible ill-defined accumulation of a modifier gene(s) in the mgR strain. Regularly performed PCR confirmed maintenance of correct genotypes during breeding (Supplemental Figure 1A; supplemental material available online with this article; https://doi.org/10.1172/JCI183558DS1). At 12 weeks of age, animals were euthanized and their mitral valves examined. Histology showed marked thickening of mitral valve leaflets and significantly increased leaflet area consistent with myxomatous mitral valve degeneration (Figure 1, A and B). In human MVP, mitral valve thickening occurs predominantly in the tips of anterior and posterior mitral valve leaflets with accumulation of ECM (16, 17). We noted similar changes in ECM composition in mgR mice with accumulation of GAGs and elastin, a relative increase in GAGs (Supplemental Figure 1B), and valve thickening of the leaflet tips (Supplemental Figure 1C). Although the number of valve cells increased, cell density decreased due to greater ECM volume (Supplemental Figure 1D). Valvular function was assessed by echocardiography and revealed MR in 46.2% (12/26) of mgR mice at 12 weeks of age compared with none in WT mice (Supplemental Figure 1E). Immunofluorescence (IF) staining showed reduced fibrillin-1 in mitral valve leaflets from mgR mice (Supplemental Figure 1F), and RT-PCR confirmed decreased Fbn1 transcription in cultured dermal fibroblasts (Supplemental Figure 1G). Mitral valve leaflets from mgR and WT mice were analyzed by bulk RNA-Seq where principal component analysis and heatmaps of differentially expressed genes (DEGs) showed clear separation by experimental condition (Figure 1C and Supplemental Figure 2A). Analysis of DEGs revealed 141 upregulated and 844 downregulated genes in mgR versus WT mice (Figure 1D). Gene Ontology (GO) query showed enrichment of terms for multiple ECM and inflammatory processes (Supplemental Figure 2B). Pathway analysis identified several signaling transduction pathways, including PI3K/Akt with the highest DEG count in addition to TGF-β and Wnt/β-catenin (Figure 1E). Accordingly, IF analysis of mitral valve tissue from mgR mice demonstrated increased phospho-PI3K (p-PI3K) and p-Smad2 (Figure 1F), as well as greater TGF-β1 ligand and less latency-associated peptide (LAP) (Figure 1G), suggesting activation of PI3K and canonical TGF-β signaling. We also explored mTOR signaling in our model since it is downstream of PI3K/Akt and confirmed increased activity by intense staining for S6 phosphorylation (p-S6) (Figure 1F). Concordant with recent studies demonstrating the importance of macrophage recruitment in MFS (6, 7), we observed a significant accumulation of CD45^+^/CCR2^+^ cells in mitral valve leaflets of mgR mice at 12 weeks of age (Figure 1H). In summary, these results demonstrate that hypomorphic mgR mice, insufficient for fibrillin-1, exhibit numerous hallmarks of myxomatous mitral valve degeneration with evidence of ECM perturbation; upregulation of TGF-β, PI3K, and mTOR signaling; and leukocytic infiltrates.

### Activation of TGF-β and mTOR signaling and leukocyte recruitment precedes myxomatous degeneration of mitral valve leaflets in mgR mice at 4 weeks of age.

**Figure 2:**
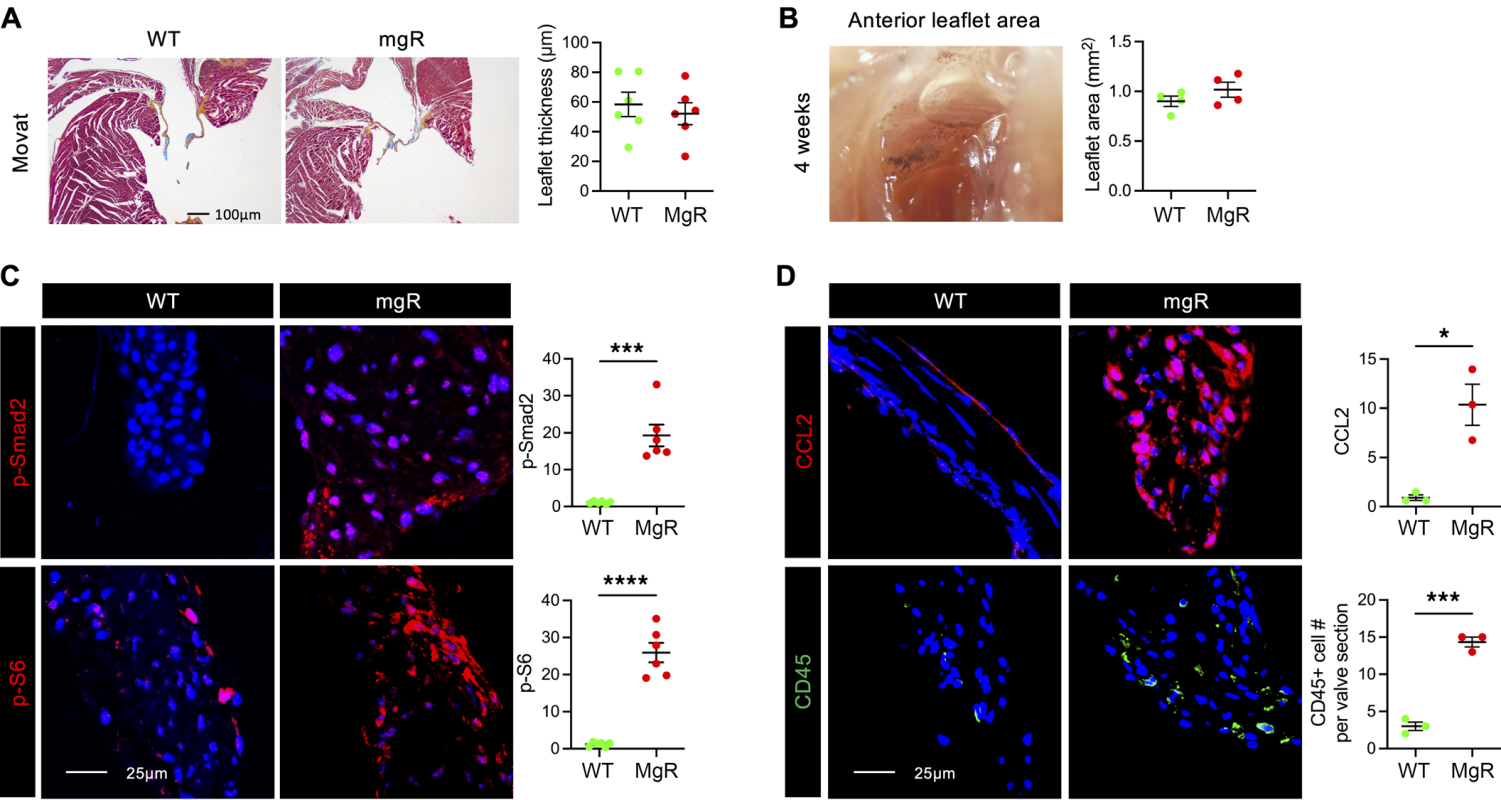
TGF-β and mTOR signaling activation and leukocyte accumulation without mitral valve leaflet degenerative changes in 4-week-old mgR mice. (**A**) Representative Movat pentachrome staining of mitral valve leaflets with maximal leaflet thickness measurement in 4-week-old WT versus mgR mice (*n* = 6). Scale bar: 100 μm. (**B**) Morphometric analysis of anterior mitral leaflet area (*n* = 4). Representative IF and mean fluorescence density measurement (AU) for (**C**) p-Smad2 and p-S6 (*n* = 6) and (**D**) CCL2 and number of CD45^+^ cells (*n* = 3). Scale bars: 25 μm. Data are represented as individual values with mean ± SEM; *P < 0.05, ***P < 0.001, ****P < 0.0001 by unpaired *t* test.

We analyzed mitral valve leaflets from the animals during postnatal development to determine the temporal relationship of signaling activation to morphological abnormalities. Although some variability was noted in leaflet thickness and mitral valve area, no significant morphological or morphometric differences were detected in mgR versus WT mice at 4 weeks of age (Figure 2, A and B). At this time point, however, there was evidence of increased p-Smad2 and p-S6, reflecting activation of TGF-β and mTOR signaling (Figure 2C). Additionally, there was elevated expression of CCL2, a ligand for CCR2, as well as increased CD45^+^ leukocytes (Figure 2D). These results demonstrate that increased TGF-β and mTOR signaling as well as monocyte/macrophage recruitment occur prior to myxomatous mitral valve degeneration, suggesting pathogenic roles. While TGF-β signaling is known to be necessary for mitral valve disease in murine models of MFS (12), a mechanistic role for mTOR signaling has not been previously described.

### Short-term rapamycin treatment prevents TGF-β signaling and leukocyte recruitment during early mitral valve disease in mgR mice at 5 weeks of age.

**Figure 3:**
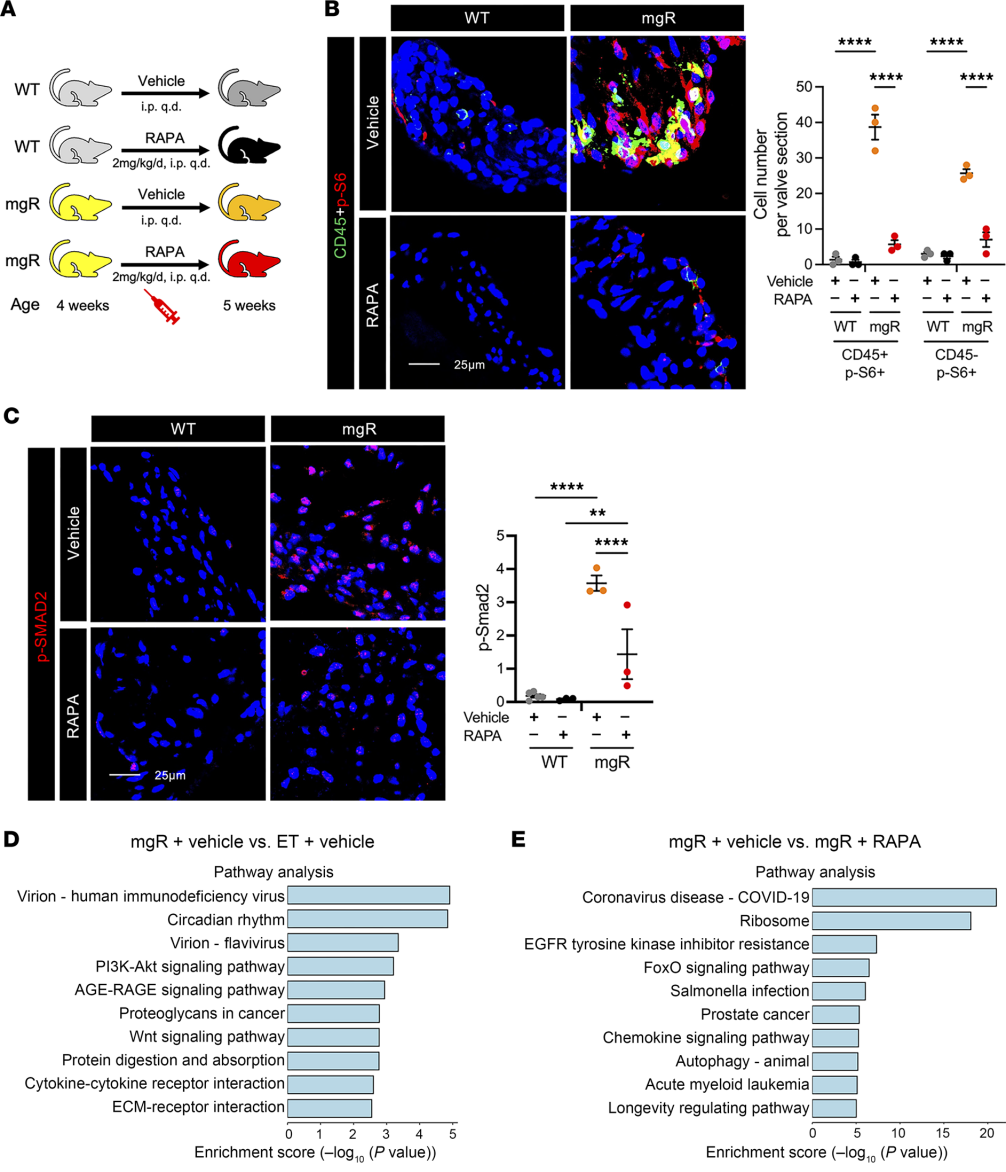
Inhibition of mTOR and TGF-β signaling and leukocyte recruitment by rapamycin during early mitral valve disease in 5-week-old mgR mice. (**A**) Experimental design: WT and mgR mice were treated with vehicle or rapamycin (RAPA) for 1 week from 4 to 5 weeks of age. Representative IF for (**B**) number of CD45^+^ and p-S6^+^ cells and (**C**) mean fluorescence density measurement (AU) for p-Smad2 in 5-week-old mice (*n* = 3–5). Scale bars: 25 μm. Pathway analysis from bulk RNA-Seq in (**D**) vehicle-treated WT versus mgR mice and (**E**) vehicle-treated versus rapamycin-treated mgR mice; input genes with adjusted *P* < 0.05. Data are represented as individual values with mean ± SEM; **P < 0.01, ****P < 0.0001 by 2-way ANOVA.

Given the rapid activation of mTOR signaling in mitral valve leaflets prior to pathologic morphological changes, we treated mgR and WT mice with the mTOR inhibitor rapamycin or vehicle control by daily injection for 1 week from 4 to 5 weeks of age (Figure 3A). Treatment resulted in effective inhibition of mTOR signaling and suppressed recruitment of CD45^+^ leukocytes into mitral valve leaflets (Figure 3B). mTOR activation was evident in both CD45^+^ and CD45^–^ cells, suggesting that mTOR signaling activation is widespread, involving leukocytes and intrinsic valve cell types. Notably, rapamycin also prevented increased TGF-β signaling prior to overt myxomatous degeneration of mitral valve leaflets (Figure 3C). Transcriptomic analysis was performed by bulk RNA-Seq from mitral valve leaflets of 5-week-old treated and untreated mice to examine drug effects. Principal component analysis and heatmaps of DEGs showed separation of groups by genotype and therapy (Supplemental Figure 3, A–E). There were 65 upregulated and 152 downregulated genes in vehicle-treated WT versus mgR mice and a larger number of 404 upregulated and 596 downregulated genes in vehicle-treated versus rapamycin-treated mgR mice. Pathway analysis suggested activation of PI3K/Akt and Wnt signaling as well as increased interactions of ECM and cytokine receptors in vehicle-treated mgR versus WT mice (Figure 3D), while GO analysis demonstrated enrichment for many inflammation and ECM-related terms (Supplemental Figure 4A). In contrast, rapamycin treatment of mgR mice for 1 week involved different pathways, including ribosomes and chemokine signaling (Figure 3E) as well as transcription, translation, and cell growth/cell cycle terms (Supplemental Figure 4B). Taken together, these results indicate that during the early phase of disease, prior to morphological evidence of tissue remodeling, mTOR signaling is activated in mitral valve leaflets. This activation coincides with increased TGF-β activity and leukocyte recruitment associated with altered expression of multiple genes and pathways, and these events are inhibited by short-term rapamycin treatment.

### Long-term rapamycin treatment prevents myxomatous mitral valve degeneration in addition to aberrant intracellular signaling and leukocytic infiltrates in advanced lesions of mgR mice at 12 weeks of age.

**Figure 4:**
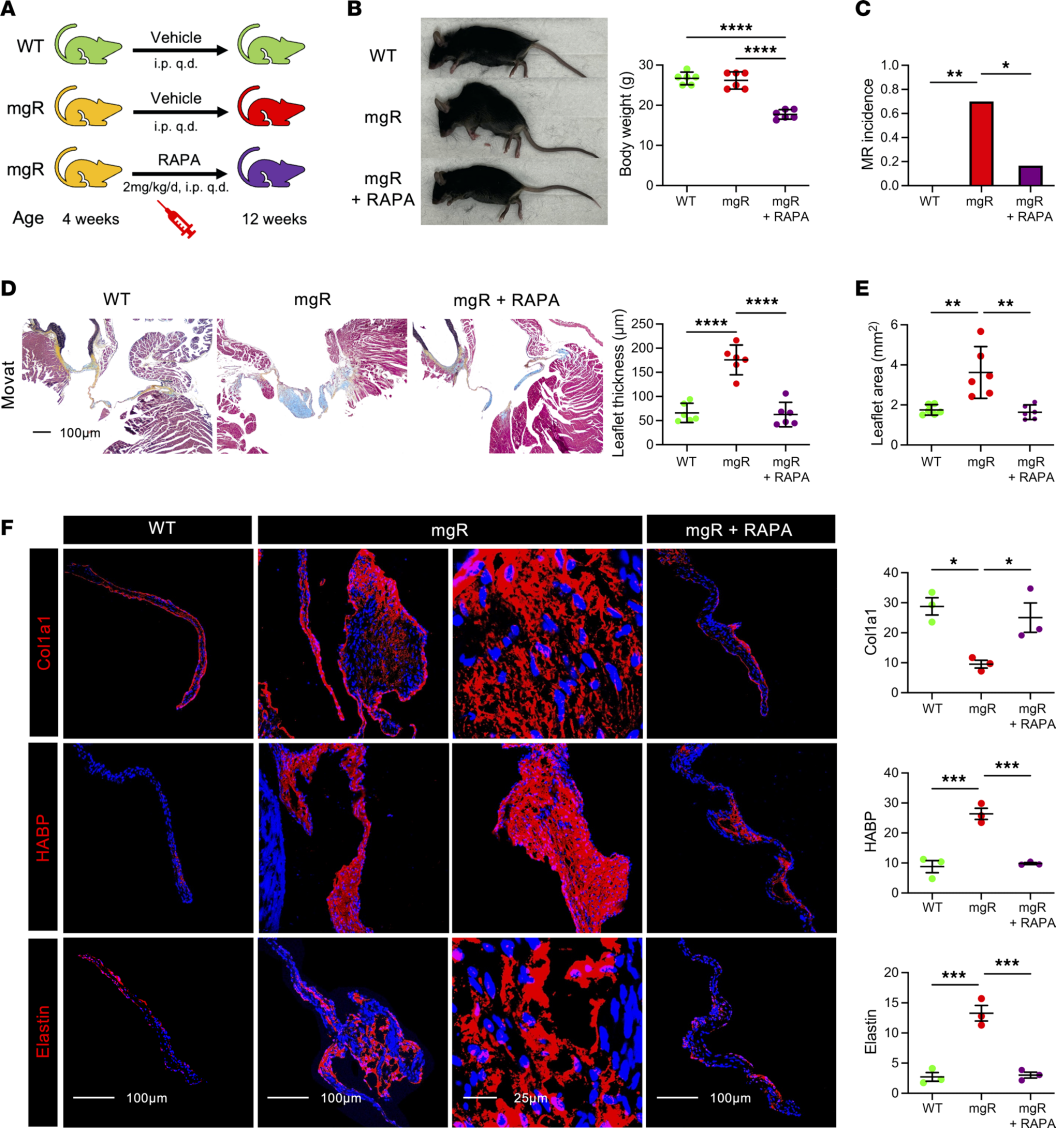
Long-term treatment with rapamycin rescues the mitral valve and skeletal phenotype in 12-week-old mgR mice. (**A**) Schema of experimental groups: WT mice were treated with vehicle and mgR mice were treated with vehicle or rapamycin for 8 weeks starting at 4 weeks of age. (**B**) Representative photographs and body weight measurement of 12-week-old mice (*n* = 6). (**C**) Incidence of MR (*n* = 6–10). (**D**) Representative Movat pentachrome staining and measurement of maximal leaflet thickness (*n* = 6). (**E**) Morphometric analysis of the anterior mitral valve leaflet area (*n* = 6). (**F**) Representative IF staining with mean fluorescence density (AU) for collagen type 1 α-1 (Col1a1), hyaluronan binding protein (HABP) for GAGs, and elastin (*n* = 3). Scale bars: 25 and 100 μm. Data are represented as individual values with mean ± SEM; *P < 0.05, **P < 0.01, ***P < 0.001, ****P < 0.0001 by (**B** and **D**–**F**) 1-way ANOVA or (**C**) Fisher’s exact test.

**Figure 5:**
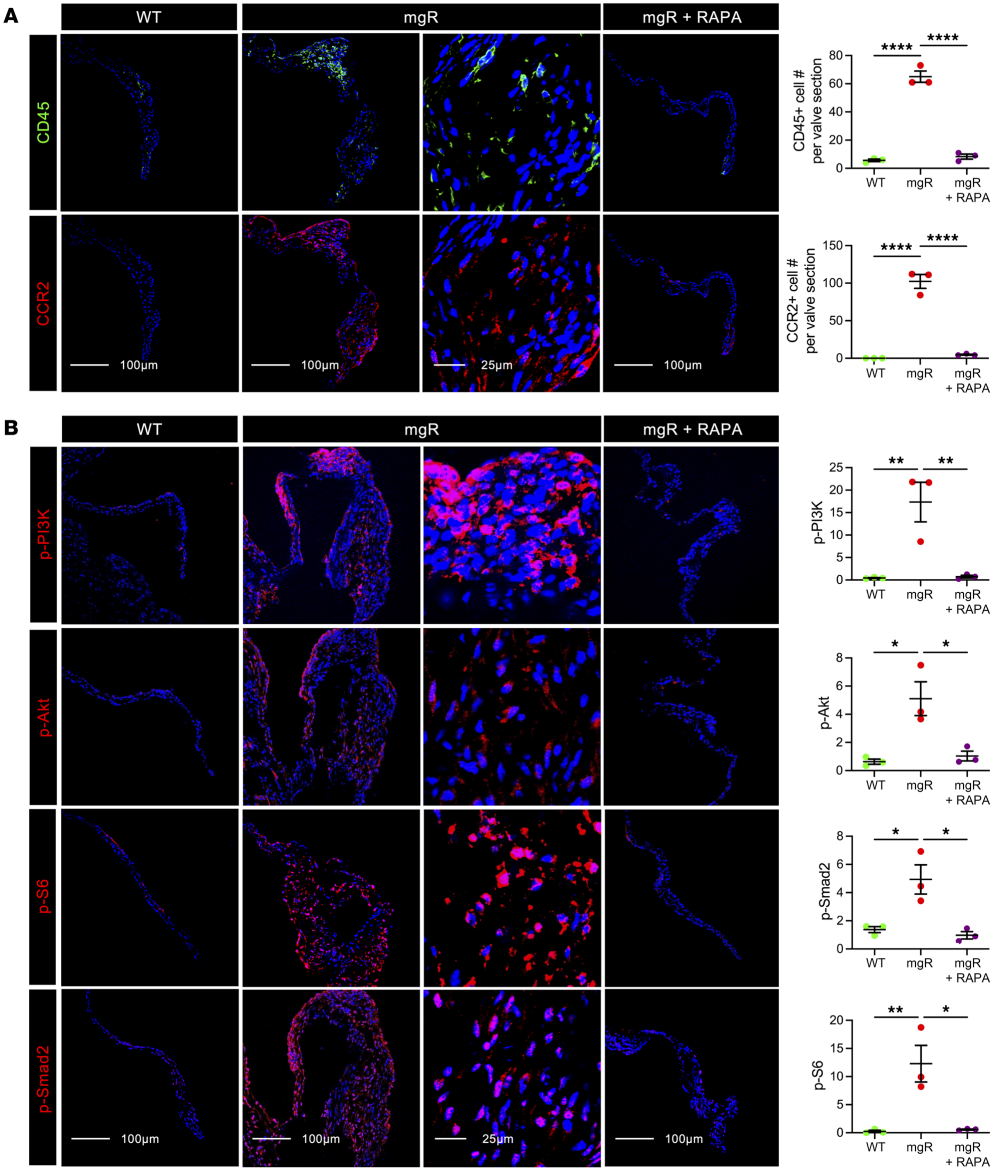
Long-term treatment with rapamycin prevents recruitment of macrophages and inhibits mTOR and TGF-β signaling in 12-week-old mgR mice. (**A**) Representative IF staining for CD45 and CCR2 with number of CD45^+^ and CCR2^+^ cells and (**B**) representative IF staining with mean fluorescence density (AU) for p-PI3K, p-Akt, p-S6, and p-Smad2 in vehicle-treated WT and vehicle- or rapamycin-treated 12-week-old mgR mice (*n* = 3). RAPA, rapamycin. Scale bars: 25 and 100 μm. Data are represented as individual values with mean ± SEM; *P < 0.05, **P < 0.01, ****P < 0.0001 by 1-way ANOVA.

To determine effects of prolonged mTOR inhibition, we treated mgR mice with rapamycin daily for 8 weeks from 4 to 12 weeks of age; controls included WT and mgR mice injected with DMSO vehicle (Figure 4A). Typical kyphosis associated with MFS was prevented, but body weight was markedly lower in rapamycin-treated animals, reflecting systemic effects of growth inhibition (Figure 4B). Incidence of MR was significantly reduced (Figure 4C) and histomorphometric analysis demonstrated no myxomatous mitral valve degenerative changes with normalization of maximal leaflet thickness and mitral valve area in mgR mice treated with rapamycin (Figure 4, D and E). The altered expression of ECM components in mitral valve leaflets of mgR mice was attenuated by rapamycin treatment (Figure 4F), and the recruitment of CD45^+^ and CCR2^+^ cells was also prevented by mTOR inhibition (Figure 5A). Long-term rapamycin treatment had broad inhibitory effects on signaling pathways silencing activation of PI3K, mTOR complex 1 ribosomal protein S6, mTOR complex 2 effector Akt (S473), and TGF-β effector Smad2 (Figure 5B) as well as Wnt/β-catenin signaling in mitral valve leaflets of mgR mice (Supplemental Figure 5). To assess if the myxomatous changes could be reversed, we initiated rapamycin treatment at 8 weeks of age and euthanized the mice at 12 weeks of age (Supplemental Figure 6A). This resulted in reduced p-S6 activity and number of CD45^+^ leukocytes, but there was no evidence of phenotype rescue by histological criteria or incidence of MR (Supplemental Figure 6, B–D). Rapamycin inhibited mTOR activity and significantly reduced recruitment of CD45^+^ cells close to WT levels (Supplemental Figure 6E). The relevance of TGF-β signaling in the mgR model was tested by treating WT and mgR mice with neutralizing TGF-β antibody or IgG1 control for 1 week starting at 4 weeks of age (Supplemental Figure 7A). Short-term TGF-β neutralization for 1 week effectively inhibited phosphorylation of Smad2 and S6 (Supplemental Figure 7, B and C). Long-term treatment from 4 to 12 weeks of age (Supplemental Figure 7D) resulted in partial rescue of the mitral valve phenotype evident by morphometric measurements (Supplemental Figure 7, E and F). In summary, these results demonstrate that while TGF-β signaling is necessary for myxomatous mitral valve degeneration, mTOR signaling is critical in disease pathogenesis, mediating recruitment of inflammatory cells and activating several pathological intracellular signaling pathways that lead to accumulation of excessive ECM characteristic of the phenotype.

### Integrins are enriched in receptor-ligand analysis of RNA-Seq of mitral valves from mgR mice.

**Figure 6:**
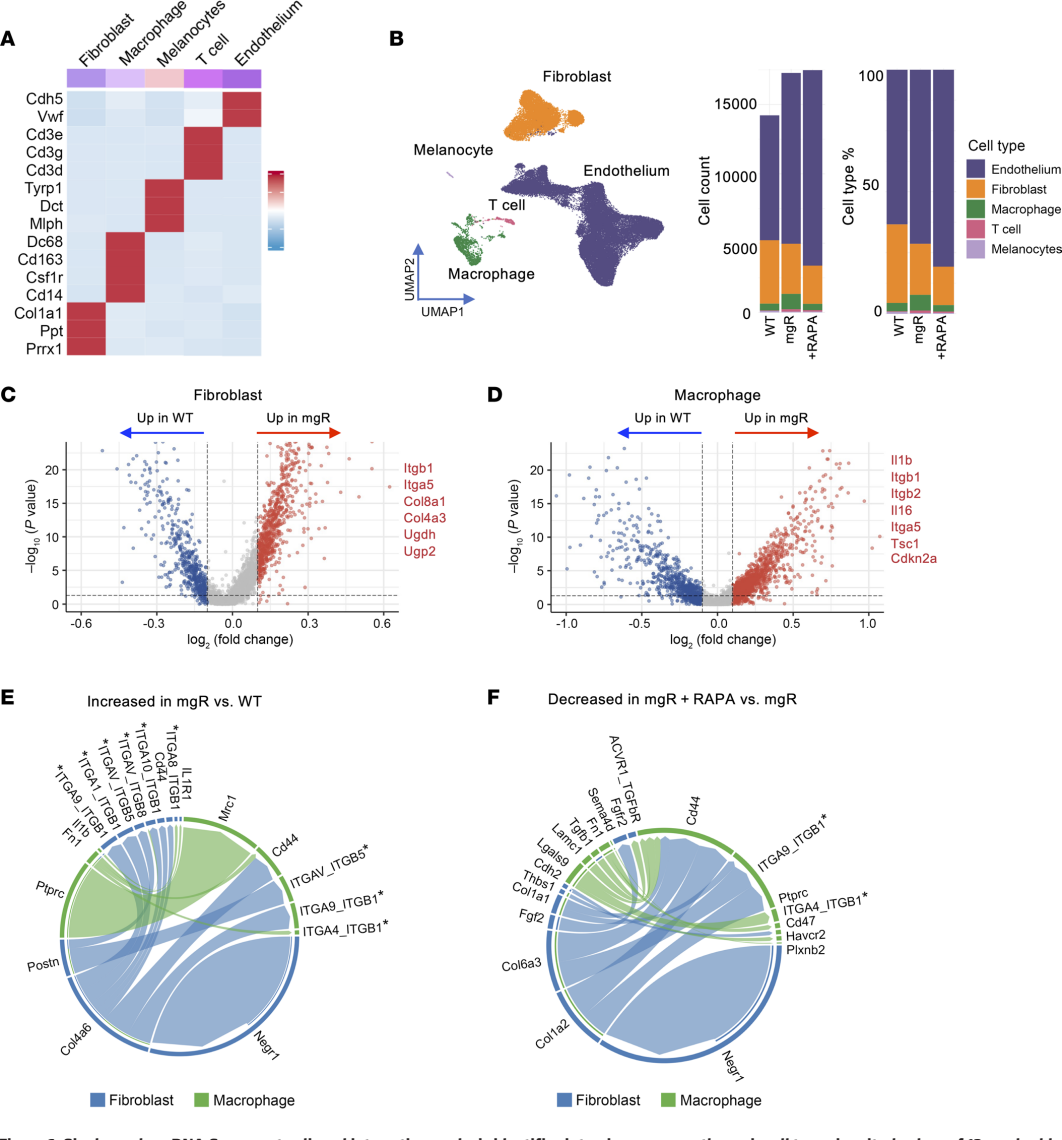
Single-nucleus RNA-Seq receptor-ligand interaction analysis identifies integrins among pathogenic cell types in mitral valves of 12-week-old mgR mice. (**A**) Heatmap of characteristic marker genes across 5 cell types. (**B**) Uniform Manifold Approximation and Projection (UMAP) plots of single-nucleus RNA-Seq with annotation of primary cell types, demonstrating proportional differences among mitral valves from WT, mgR, and rapamycin-treated mgR (+RAPA) mice at 12 weeks of age. Volcano plot showcasing DEGs of (**C**) fibroblasts and (**D**) macrophages in mitral valve leaflets from WT and mgR mice, with selected DEGs labeled. Genes highlighted in red are upregulated in mgR, while those in blue are upregulated in WT. Chord diagrams illustrating the strength of receptor-ligand signaling interactions among macrophages and fibroblasts in (**E**) mgR versus WT (reference) mice and (**F**) rapamycin-treated mgR versus mgR (reference) mice; *integrins identified as receptors.

To gain further insight into mechanisms associated with mTOR-mediated myxomatous valve degeneration, we performed 2 sets of transcriptomic analysis using 12-week-old mice: (a) single-cell RNA-Seq analysis using mitral valve leaflets from WT and mgR mice and (b) single-nucleus RNA-Seq using WT, mgR, and mgR mice treated with rapamycin for 8 weeks. Characteristic markers were used to identify 5 primary cell types: fibroblasts, macrophages, endothelium cells, T cells, and melanocytes (Figure 6A and Supplemental Figure 8A). The number of cells was increased in myxomatous mitral valves, aligning with histological analysis, with proportional increases in macrophages and decreases in fibroblasts in mgR compared with WT mice (Figure 6B and Supplemental Figure 8, B and C). The RNA yield was higher for single-nucleus RNA-Seq, including a disproportionately higher number of endothelial cell markers compared with single-cell RNA-Seq, and expected based on histological changes. This is likely related to the difference in protocols for single-cell and nuclear RNA extraction. Variance of cellular components should be interpreted cautiously since the yield may also depend on different incorporation of cell types within the ECM; for example, fibroblasts may be more difficult to isolate than superficial endothelial cells. We focused subsequent single-nucleus RNA-Seq analysis on fibroblasts and macrophages demonstrating marked differences in gene expression within cell types when comparing WT and mgR mice (Figure 6, C and D). Gene set enrichment analysis comparing fibroblasts in mgR and WT mice showed increases in pathway expression for ECM organization, cell adhesion, chemokine production, and leukocyte activation (Supplemental Figure 8D). In macrophages, the analysis highlighted pathways associated with regulation of cell death, apoptosis, and adhesion processes (Supplemental Figure 8E). Receptor-ligand analysis for fibroblasts and macrophages showed increased signaling interactions in mgR versus WT mice (Figure 6E). Upregulated ligands included Fn1 (encoding fibronectin) and Ptprc (encoding CD45) in macrophages and Postn, Col4a6, and Negr1 in fibroblasts. The predominant upregulated receptors for both fibroblasts and macrophages included the integrin superfamily, especially β_1_ integrin (Figure 6E). Rapamycin treatment for 8 weeks resulted in decreased receptor-ligand interactions including β_1_ integrin as well as TGF-β1 and ECM components (Figure 6F). In summary, transcriptomic analysis identified a marked increase in receptor-ligand interactions, particularly between integrins and their ligands, suggesting that cell-cell and cell-ECM adhesion as well as mechanosignaling are affected in mitral valve tissue from mgR mice.

### Integrins are required for mTOR signaling and development of the mitral valve phenotype in mgR mice.

**Figure 7:**
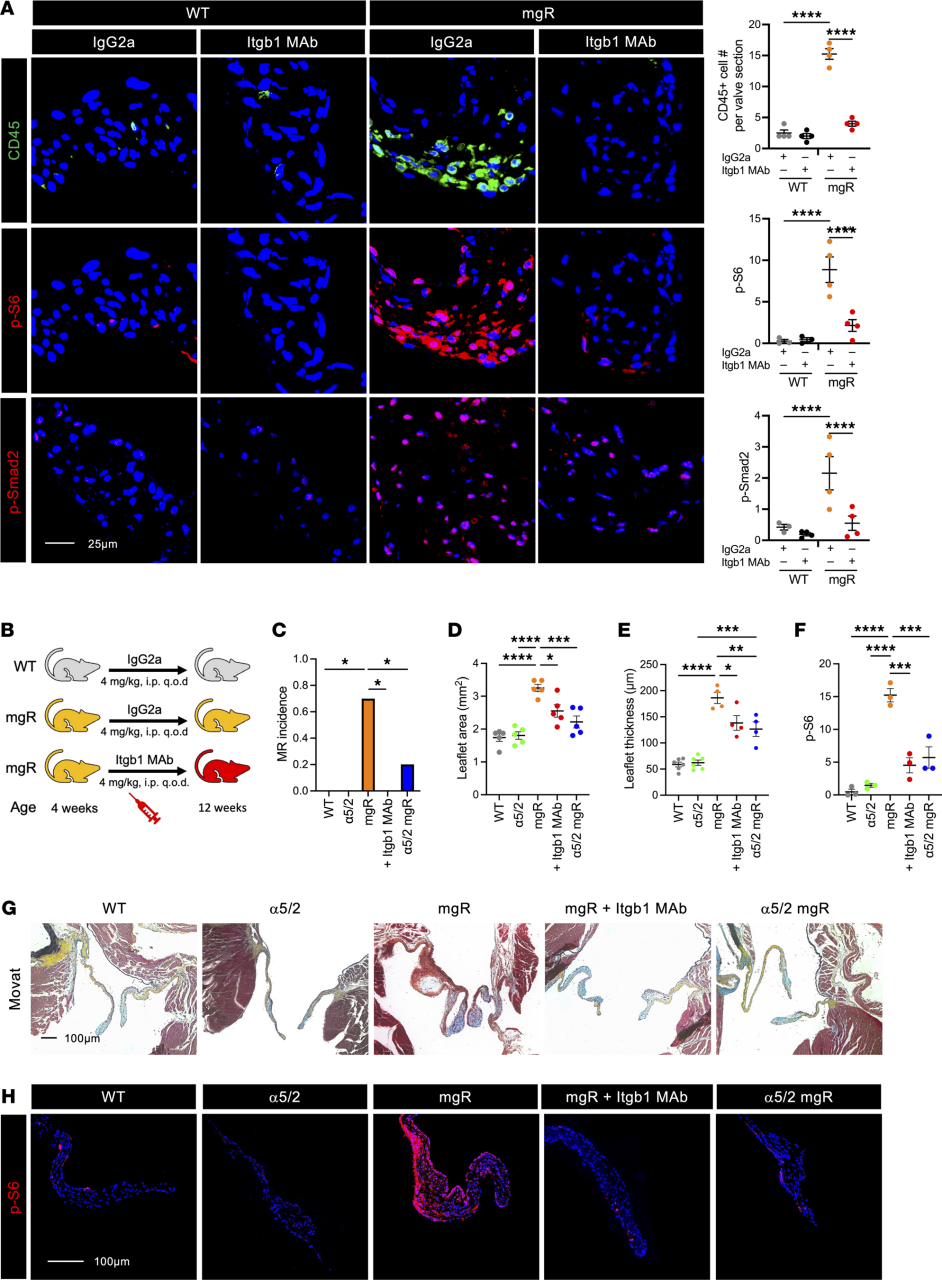
Integrin signaling is required for mTOR and TGF-β activation evident by interference via β_1_ integrin neutralizing antibody (Itgb1 mAb) treatment and integrin α5/2 chimeric mutation with partial rescue of mgR mitral valve phenotype. (**A**) Representative IF and number of CD45^+^ cells (*n* = 4) and mean fluorescence density (AU) for p-S6 (*n* = 3–4) and p-Smad2 (*n* = 3–4) in 5-week-old WT and mgR mice treated with either IgG2a control or Itgb1 mAb for 1 week starting at 4 weeks of age. Scale bar: 25 μm. (**B**) Schema depicting serologic neutralization experimental design in which mgR mice were treated with Itgb1 mAb for 8 weeks starting at 4 weeks of age with mitral valves analyzed at 12 weeks of age. (**C**) Incidence of MR (*n* = 10) and (**D**) morphometric analysis of anterior mitral valve leaflet area (*n* = 5). (**E**) Measurement of maximal leaflet thickness (*n* = 4–6) and (**F**) mean fluorescence density (AU) for p-S6 (*n* = 3) in 12-week-old WT, α5/2, mgR, mgR + Itgb1 MAb, and α5/2 mgR mice. (**G**) Representative Movat pentachrome staining of long-axis sections of mitral valve leaflets and (**H**) representative IF of p-S6 in 12-week-old WT, α5/2, mgR, mgR + Itgb1 MAb, and α5/2 mgR mice. Scale bars: 100 μm. Data are represented as individual values with mean ± SEM; *P < 0.05, **P < 0.01, ***P < 0.001, ****P < 0.0001 by (**D**–**F**) 1-way or (**A**) 2-way ANOVA or (**C**) Fisher’s exact test.

β_1_ Integrin was increased in mitral valves from mgR mice at both 4 and 12 weeks of age (Supplemental Figure 9A). To investigate the role of β_1_ integrin in early disease, we administered neutralizing mAb targeting β_1_ integrin (Itgb1) for 1 week in mgR mice from 4 to 5 weeks of age (Supplemental figure 9B), resulting in reduced mTOR and TGF-β activity as well as leukocyte recruitment in 5-week-old mgR mice (Figure 7A). We further treated mgR mice with Itgb1 mAb for 8 weeks, from 4 to 12 weeks of age (Figure 7B), and analyzed a unique chimeric integrin mouse model where the α_5_ integrin (Itga5) cytoplasmic domain is replaced with that of α_2_ integrin, resulting in resistance to atherosclerosis and decreased inflammatory activation (18). The integrin α5/2 chimera mice were bred with mgR heterozygotes to generate double homozygous mutants, denoted as α5/2 mgR (Supplemental Figure 9C) (19). At 12 weeks of age, 70% of mgR mice and 20% of α5/2 mgR mice demonstrated MR, while WT, α5/2, and Itgb1 mAb-treated mgR mice did not develop MR (Figure 7C). The α5/2 mice had normal mitral valve pathology compared with WT mice. Long-term treatment of mgR mice with Itgb1 mAb resulted in significant, but incomplete, rescue of the mitral valve phenotype, and similar partial phenotypic rescue was seen in 12-week-old α5/2 mgR mice (Figure 7, D, E, and G). p-S6 was effectively inhibited by long-term treatment with Itgb1 mAb, and mTOR activity was also decreased in α5/2 mgR mice (Figure 7, F and H). Concurrent with fibronectin identified as a primary integrin ligand elevated in receptor-ligand analysis, the levels of β_1_ integrin, α_5_ integrin, and fibronectin were increased in 12-week-old mgR mice compared with WT mice, which were all attenuated following long-term treatment with rapamycin in mgR mice (Supplemental Figure 10). These results suggest that β_1_ integrin receptor–mediated signaling, in particular that of α_5_β_1_, is necessary for activation of both mTOR and TGF-β signaling in myxomatous mitral valve disease of fibrillin-1–deficient mgR mice.

### Conserved transcriptome signature in human mitral valve disease specimen with activation of mTOR signaling and increased integrin expression.

**Figure 8:**
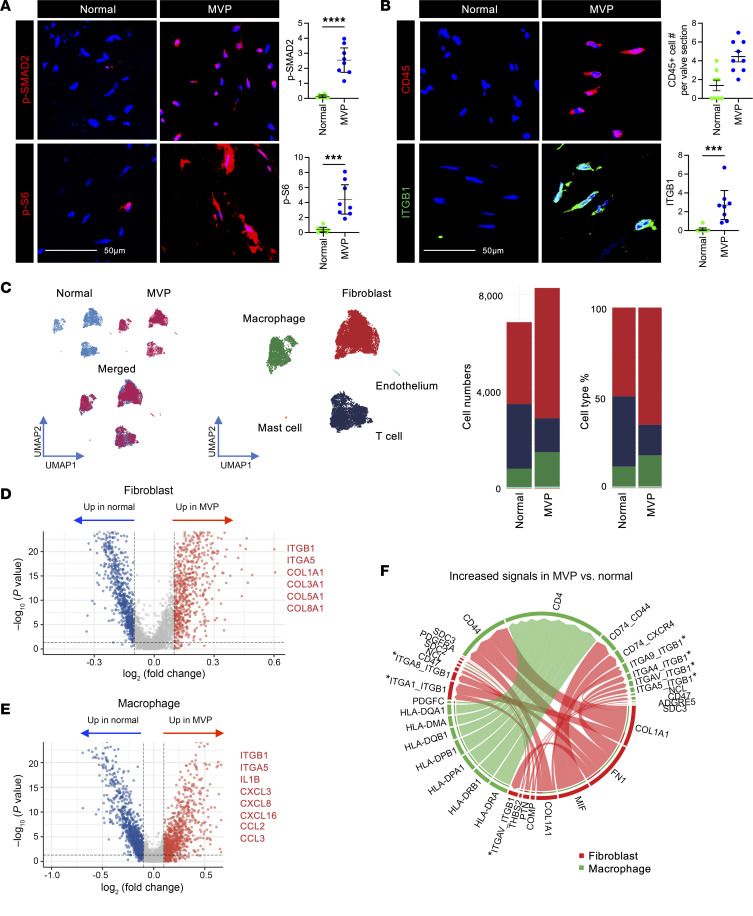
Evidence of TGF-β and mTOR signaling activation and β_1_ integrin expression with conserved transcriptome signature in human myxomatous mitral valve degeneration. Representative IF and mean fluorescence density (AU) in human normal mitral valves and MVP specimens for (**A**) p-Smad2 and p-S6 (*n* = 8), and (**B**) CD45 (*n* = 8–9) and β_1_ integrin (ITGB1) (*n* = 8–10). Scale bars: 50 μm. (**C**) Uniform Manifold Approximation and Projection (UMAP) plots of single-cell RNA-Seq with annotation of primary cell types, demonstrating proportional differences between mitral valves from normal and MVP specimen (*n* = 4–6). Volcano plot showcasing DEGs of (**D**) fibroblasts and (**E**) macrophages from normal and MVP valves, with selected DEGs labeled. Genes highlighted in red are upregulated in MVP, while those in blue are upregulated in normal valves. (**F**) Chord diagram illustrating the strength of receptor-ligand signaling interactions among macrophages and fibroblasts in MVP versus normal as reference; *integrins identified as receptors. Data are represented as individual values with mean ± SEM; ***P < 0.001, ****P < 0.0001 by unpaired *t* test.

To determine the translational relevance of our findings in mice, we examined human specimens of MVP from surgical repair or replacement and normal mitral valves from organ donors (Supplemental Table 1). MVP tissue demonstrated activation of TGF-β and mTOR signaling with increased staining for pSMAD2 and p-S6, respectively, as well as increased CD45^+^ cells, β_1_ integrin, and fibronectin (Figure 8, A and B, and Supplemental Figures 11 and 12A). Single-cell RNA-Seq analysis of mitral valve leaflets from normal and diseased valves identified 5 primary cell types using standard markers (Supplemental Figure 12B). This included the same cell types as seen in murine mitral valves except instead of murine melanocytes, human valves contained a small number of mast cells. Fibroblasts were in much higher abundance in human than in mouse valve tissue (Figure 8C). This may be due to differences in yield of cell extraction in thicker human tissues compared with thinner mitral valve leaflets in the mouse or a representation of the more chronic condition of MVP in humans. There were marked and distinct differences in the expression profiles and pathway analyses between normal and MVP conditions in both fibroblasts and macrophages (Figure 8, D and E). Gene set enrichment analysis revealed increased pathway expression in MVP compared with normal fibroblasts for ECM organization, immune response, cytokine stimulation, and cell death (Supplemental Figure 12C) as well as in MVP compared with normal macrophages for regulation of cell death, response to cytokine, and cell communication (Supplemental Figure 12D). Consistent with observations in mgR mice, receptor-ligand analysis of macrophages and fibroblasts revealed an increased number and strength of interactions in MVP tissue compared with normal tissue. The integrin superfamily emerged as the predominant receptor group, while ligands primarily included collagen subtypes and fibronectin (Figure 8F). These results suggest that activation of mTOR and TGF-β signaling, inflammation, and increased integrin expression are relevant to MVP pathobiology in humans comparable to that observed in mgR mice.

## Discussion

We used genetically modified mgR mice expressing low levels of fibrillin-1 that developed severe myxomatous mitral valve degeneration postnatally to study the pathogenesis of MVP. Increased TGF-β and mTOR signaling and macrophage recruitment preceded changes in mitral valve morphology, and these early changes were attenuated by short-term rapamycin therapy; long-term inhibition of mTOR fully rescued the mitral phenotype, while inhibition of TGF-β signaling resulted in partial rescue. Receptor-ligand interaction analysis identified integrins as key receptors linking pathogenic cell types. Short-term treatment with neutralizing antibody against β_1_ integrin in mgR mice decreased TGF-β signaling, mTOR signaling, and leukocytic infiltrates, while long-term treatment or crossbreeding with integrin α5/2 chimeric mice resulted in incomplete rescue of the mitral valve phenotype. Increased mTOR signaling with associated increased integrin expression and a comparable transcriptomic signature were observed in human MVP. Together, these findings support that integrin-mediated mTOR signaling drives TGF-β overactivation, inflammation, and myxomatous mitral valve degeneration, and we propose mTOR inhibition as a possible medical therapy for MVP.

The *Fbn1^C1041G/+^* MFS model has a well-described mitral valve phenotype, while mitral valve pathology in the hypomorphic mgR model, better known for developing aortic aneurysm and dissection, has not been previously reported (6, 7, 9, 11, 15). The *Fbn1^mgR^* mutation contains a neo cassette insertion into intron 18 without loss of coding sequence, resulting in severely reduced *Fbn1* mRNA expression in homozygous mgR compared with WT mice. Deficiency of fibrillin-1 caused severe myxomatous pathological changes of the mitral valve in vivo by 12 weeks of age. The changes observed in mitral valves, such as leaflet lengthening, tip thickening, marked accumulation of GAGs, and overall increase in collagen and elastin, are similar to those described in human MFS subjects, of which 25% had clinical evidence of myxomatous mitral valve phenotype (20), as well as in primary MVP, where the majority of patients have no identifiable genetic mutation or syndrome (21). The morphological changes noted within 3 months of age in mgR mice thus provide an appropriate model to study the pathogenesis of MVP, identify disease mechanisms, and test potential therapies.

Transcriptomic analysis of mitral valve leaflet tissue from mgR mice implicated upregulation of several signaling pathways, including TGF-β, confirmed by phosphorylation of Smad2, as well as PI3K/Akt with evidence of mTOR activation by p-S6. Activation of the TGF-β canonical pathway in mitral valves of *Fbn1^C1041G^* mice is well established (11), and TGF-β is necessary and sufficient for ECM production in cultured human mitral valve tissue, supporting a role for TGF-β in myxomatous degeneration of sporadic MVP (22). Like descriptions in *Fbn1^C1041G^* mice, we noted upregulation of canonical TGF-β signaling and evidence of reduced LAP associated with increased active TGF-β1 in mitral valves from mgR mice. The importance of TGF-β in disease pathogenesis was demonstrated by inhibition of p-Smad2 and p-S6 signaling within 1 week of neutralizing TGF-β antibody and partial rescue of the mitral valve phenotype in mgR mice after 8 weeks of treatment. These results differ from a prior study focusing on aneurysm formation in mgR mice where TGF-β neutralization, initiated on postnatal day 16, exacerbated aneurysm progression and early mortality, suggesting genetic drift in our mgR colony with a less severe aortic phenotype (23). This is a potential limitation of our study but does not reduce the relevance of the severe mitral phenotype observed in the mgR substrain and littermate controls described herein.

The extensive morphological changes observed in ECM composition of both older mgR mice and myxomatous mitral valve tissue in patients with advanced disease likely represent end-stage pathological changes where signaling pathway analysis may be obscured by secondary events. To identify likely pathogenic mechanisms rather than secondary processes, we analyzed mitral valve leaflets at 4 weeks of age before substantial morphological abnormalities manifested. At this young age, prior to ECM accumulation, there was activation of TGF-β and mTOR signaling associated with increased CCL2 expression and CCR2^+^ leukocyte recruitment. Pro-inflammatory macrophage accumulation is necessary for disease development in *Fbn1^C1041G/+^* mice, and macrophages are present in mitral valves of Axin2-deficient mice and human MVP (7, 13, 24). Aortopathy in mgR mice also includes inflammatory fibroproliferative responses and accumulation of activated macrophages within the aortic wall (15). Whether signaling perturbations precede or occur concurrently with macrophage infiltration remains unknown. mTOR signaling is necessary for many immune responses and influences several aspects of leukocyte function, including recruitment and activation of macrophages (25). It is therefore not unexpected that mTOR has a role in myxomatous mitral valve degeneration.

Identification of increased mTOR activity prompted us to study the effects of rapamycin treatment on signaling as well as secondary processes. Short-term (1 week) treatment in young mice inhibited mTOR signaling in leukocytes and intrinsic valvular cells and decreased recruitment of leukocytes to leaflet tissue. Short-term treatment also affected processes related to cell cycle and proliferation, as expected with a prominent regulatory role of mTOR in cell growth and proliferation (26). Long-term (8 weeks) treatment with rapamycin resulted in complete rescue of the mitral valve phenotype in mgR mice. The effects of rapamycin were widespread, resulting in inhibition of mTOR complexes 1 and 2 in addition to TGF-β and Wnt/β-catenin signaling. Rapamycin primarily targets mTORC1, preventing binding of regulatory-associated protein of mTOR (raptor) to mTOR, thereby blocking downstream phosphorylation of S6K1 and other direct substrates (27). Inhibition of both mTOR and TGF-β canonical signaling by either rapamycin or TGF-β–neutralizing antibody supports the presence of considerable crosstalk between signaling pathways, including mTOR, TGF-β, and Wnt/β-catenin, although biological effects are context specific (28–30). End-stage consequences of ECM accumulation and disorganization in mgR mice were also rescued by long-term rapamycin treatment. Disease pathogenesis starts at an early age, and there appears to be a threshold for rescue. Later treatment with rapamycin starting at 8 weeks of age for 4 weeks did not rescue the mgR mitral valve phenotype, as evident by histology and echocardiography, despite effective inhibition of mTOR signaling and inflammation.

Our study demonstrated ubiquitous effects of mTOR inhibition, with rapamycin affecting multiple signaling pathways, macrophage recruitment, and ECM turnover in the mitral valve, maintaining normal leaflet morphology and function. Rapamycin treatment attenuates aneurysm formation and increases lifespan in mgR mice, but the mechanisms have not been defined (31, 32). Both TGF-β and PI3K/Akt/mTOR signaling pathways are involved in transition of valvular interstitial cells to a secretory phenotype in an in vitro canine model where mTOR inhibition attenuates cell senescence (33). Whether mTOR inhibition can effectively modulate other mouse models of myxomatous mitral valve degeneration, such as *Fbn1^C1041G^* or Axin2 deficiency, remains to be tested.

There is no clear evidence for direct interaction between fibrillin-1 and mTOR, but integrins are potential intermediary candidates. Using single-cell and single-nucleus RNA-Seq data, we explored receptor-ligand interaction profiles between fibroblasts and macrophages in mgR versus WT mice and observed complex and overlapping interactions within and between cell types where the integrin superfamily were the predominant receptors for several ligands. Fibrillin-1 contains Arg-Gly-Asp (RGD) motifs that directly bind to certain integrins and is related to various processes, including mechanosensing and signal transduction (34–36). β_1_ Integrin is expressed at high levels in mitral valves of mgR mice at 4 weeks of age, suggesting that fibrillin-1 deficiency induces compensatory increases in integrin levels. PI3K/Akt/mTOR exists at the junction of integrin-mediated ECM–cell interactions and mechanotransduction related to cancer progression (37, 38). It is possible that abnormal microfibrils resulting from early fibrillin-1 deficiency disrupt integrin-mediated mechanosignaling within the ECM. Using neutralizing β_1_ integrin mAb in vivo (39), we demonstrated that β_1_ integrin is required for early mTOR activation and leukocyte recruitment in mitral valve disease as well as the later development of ECM changes characteristic of mitral valve degeneration. The importance of particular integrin signaling in this model is evident by reduced p-S6 and partial phenotype rescue in the chimeric integrin α5/2 mgR mouse model (19). The importance of integrin is further supported by increased expression in human myxomatous mitral valve tissues, the association of juvenile idiopathic arthritis and MVP with *FBN1* mutations involving integrin binding sites (40), and the demonstration that mechanical stretching can stimulate Akt/mTOR signaling via β_1_ integrin, resulting in ECM production (41). Upregulated integrin signaling in smooth muscle cells is important in aortic aneurysm formation in *Fbn1^C1041G/+^* and mgR mice via fibronectin (19, 42). Integrins and fibronectin were increased in the mgR mitral valve phenotype, so similar mechanisms are likely involved in myxomatous MVP through fibroblast-mediated interactions with ECM. Fibronectin is necessary for correct fibrillin-1 deposition, and it is possible that increased fibronectin in MFS and in aortic aneurysm of mgR mice reflects a compensatory response to fibrillin-1 deficiency (19, 43). While active TGF-β is required for pathogenesis, the relative contribution of fibronectin, which also binds LAP (44) to fibrillin-1–mediated sequestration of latent TGF-β, is unclear, but our findings challenge the prevailing concept that increased bioavailability of TGF-β is a direct consequence of fibrillin-1 deficiency in MFS. Rather, a combination of fibrillin-1 deficiency and altered mechanosignaling mediated by integrin receptors and its ligands results in exaggerated downstream signaling cascades, including the PI3K/Akt/mTOR pathway, promoting cell proliferation, ECM production, metabolism, and macrophage recruitment. The canonical TGF-β signaling is rather one of many activated signaling pathways involved, while feedback loops and crosstalk with mTOR at its center mediate the pathogenesis of myxomatous mitral valve disease. Inhibition of mTOR with rapamycin deactivates multiple processes rescuing the mgR mitral valve phenotype, demonstrating clinical therapeutic potential for mTOR inhibitors. However, there is insufficient evidence to establish a linear sequence connecting fibrillin-1, integrin, and mTOR signaling to TGF-β activation and leukocyte recruitment, culminating in myxomatous degeneration of the mitral valve.

Several mTOR inhibitors, including sirolimus, temsirolimus, and everolimus, are approved for clinical application as immunosuppressive agents in organ transplant recipients and as noncytotoxic agents for the treatment of certain cancers (45). Low-dose rapamycin is well tolerated, and there are several ongoing clinical trials studying prophylactic rather than therapeutic applications, such as effects on ovarian aging (46), skeletal muscle mass (47), and longevity (48). Integrin-targeting, small-molecule or monoclonal antibody drugs are also available but are less used in clinical practice, and several trials are underway in cancer therapy, multiple sclerosis, and fibrotic diseases (49).

In conclusion, a central role of integrin-mediated mTOR signaling in the pathogenesis of myxomatous mitral valve disease was demonstrated by rescue of the phenotype with rapamycin in the mgR mouse model. The associated quiescence of inflammatory cell activation and several pathological intracellular signaling pathways demonstrates the critical role that mTOR plays and, along with evidence of mTOR activation and preserved transcriptomic signature in human specimen of MVP, provides an opportunity to test if clinical application of mTOR inhibition is feasible to modulate the progression of mitral valve disease.

## Methods

### Sex as a biological variable.

Female and male mgR mice developed myxomatous mitral valve degeneration. Only male mice were used for experimental comparisons to obtain mitral valve area measurements in a uniform population and minimize possible confounding factors affecting transcriptomic analyses and pathway interpretations.

### Mice.

C57BL/6J mice (stock no. 000664; purchased from The Jackson Laboratory) and *Fbn1^mgR/mgR^* mice (stock no. 005704; available from The Jackson Laboratory), a gift from Francesco Ramirez (Icahn School of Medicine at Mount Sinai, New York, New York, USA), were used and denoted as WT and mgR, respectively. The mgR colony was maintained in our animal facility since 2017 and was initially maintained through homozygous matings for several generations. Then, heterozygotes were bred to a C57BL/6J background for >6 generations before initiation of the study, and the colony was continued with heterozygote-to-C57BL/6J matings. Since the separation from founder animals exceeds 20 generations, our colony constitutes a unique substrain with early mortality from aortic rupture less than previously described (15), suggesting genetic drift in which severity of the original mgR aortic phenotype is reduced. For experiments, *Fbn1^mgR/+^* heterozygotes were interbred to yield *Fbn1^mgR/mgR^* mice and littermate *Fbn1^+/+^* controls. Integrin α5/2 chimera mice, a gift from Martin A. Schwartz (Yale School of Medicine, New Haven, Connecticut, USA), denoted as α5/2, were bred with Fbn1*^mgR/+^* heterozygotes to generate double homozygous mutants, denoted as α5/2 mgR mice (19). Mice were euthanized at various ages for analysis. All mouse strains used in this study were in the C57BL/6J background.

### Animal treatment.

Animals were treated with (a) rapamycin (Calbiochem) at 2 mg/kg/d i.p. every other day (q.o.d.) versus DMSO vehicle alone, (b) β_1_ integrin (CD29) monoclonal antibody (BioXCell BE0232) at 4 mg/kg/d i.p. q.o.d. versus IgG2a isotype control (BioXCell BE0089), and (c) TGF-β mAb (BioXCell BE0057) at 250 μg i.p. q.o.d. versus IgG1 isotype control (BioXCell #BE0083) for various durations as described, including a final dose 6 hours before euthanasia.

### In situ and ex vivo examination.

After euthanasia, the chest cavity was opened widely and the hearts were flushed with normal saline, excised, and fixed in 4% paraformaldehyde overnight at 4°C. The mitral valve was exposed through a longitudinal incision through the left ventricle. A SZX16 stereoscopic microscope with and Olympus camera was used to obtain in situ images of the mitral valve. Morphometry was performed in triplicate from calibrated digital images using ImageJ software (http://rsbweb.nih.gov/ij/) after outlining the edges of the mitral valve. The height was measured from the highest to the lowest points of the mitral valve. The width was measured from the far left to the far right of the valve.

### Ultrasound.

Transthoracic echocardiographic images of the heart were obtained in lightly isoflurane-anesthetized animals using high-resolution ultrasound (Vevo 2100; FUJIFILM VisualSonics) with a 40 MHz frequency linear array transducer to detect the presence of MR by an experienced sonographer.

### Histomorphometry and immunohistochemistry.

Serial 7 μm thick sagittal sections were cut from formalin-fixed, paraffin-embedded specimens. Histological and immunohistochemistry stains were performed by Yale’s Research Histology Laboratory using standard techniques. ECM components were quantified from histological images using ImageJ for monochromatic stains or a custom color segmentation algorithm for polychromatic stains; relative content was calculated as a percentage of valve leaflet area.

### Fluorescence microscopy.

Mouse hearts were fixed overnight in 4% paraformaldehyde at 4°C and either embedded in paraffin or transferred to 15% sucrose for 6–8 hours at 4°C followed by embedding in OCT compound (Tissue-Tek). Human myxomatous mitral valve specimens were promptly placed in normal saline at 4°C and processed by embedding in OCT compound within 1 hour of excision. Normal human mitral valves were harvested from tissue donors immediately after excision of the heart. Tissue blocks were sectioned at 7 μm thickness. For paraffin sections, the slides were dewaxed in xylene and boiled for 30 minutes in citrate buffer (Vector Laboratories) for antigen retrieval. Primary antibodies for labeling were from (a) Cell Signaling Technology: p-S6 (4858), β-catenin (8480), p-PI3K (4228), p-Akt (4060), p-Smad2 (18338); (b) Abcam: elastin (Ab21610), Ccr2 (Ab216863), collagen I (Ab34710), TGF-β1 (Ab92486), Ccl2 (Ab25124), Wif1 (Ab186845), Wisp2 (Ab38317), α_5_ integrin (Ab150361), fibronectin (Ab2413); (c) R&D Systems: CD45 (AF114), LAP (MAB7666); (d) Millipore: HABP (385911), β_1_ integrin (MAB1997); and (e) Invitrogen: isotype-matched, irrelevant IgG. Detection of unconjugated primary antibodies was visualized with Alexa Fluor 488–, 568–, or 647–conjugated IgG (Invitrogen). ImageJ software was used to determine the sum of the values of pixels within each valve section divided by area, resulting in mean fluorescence density value as AU used to quantify IF staining or average number of positive stained cells for specific antibodies.

### PhenoCycler-Fusion (CODEX).

Tissues were embedded in OCT and cut into 7 μm thick sections. The prepared tissue slices were subjected to staining using DNA-linked antibodies, followed by fluorophore cycling to facilitate multiplexed imaging. Image acquisition occurred over multiple rounds utilizing a microscope, with parameters fine-tuned for each specific fluorophore.

### Bulk RNA-Seq.

Total RNA was isolated from mitral valve leaflets after careful excision to ensure no myocardial contamination occurred. The SMART-Seq v4 Ultra Low Input RNA kit (Takara) was utilized for high-quality RNA-Seq. Quality was assessed by a NanoDrop spectrophotometer (Thermo Fisher Scientific) and an Agilent 2100 Bioanalyzer. Next-generation, whole-transcriptome sequencing was performed using a NovaSeq 6000 System (Illumina) at the Yale Center for Genome Analysis. RNA-Seq reads were aligned to a reference genome using the STAR short-read aligner software (50). Gene expression was quantified using RSEM with GENCODE annotation. Read counts were normalized using the trimmed mean of M-values method, and DEGs were identified using the R package DESeq2 (v3.17) (51). DEGs between experimental groups with adjusted *P* ≤ 0.05 were determined for the GO biological processes, cellular component, and molecular function categories as well as by pathway analysis with clusterProfiler, the Pathview package, and Metascape (https://metascape.org/). Clustering was unsupervised. Enriched terms were considered statistically significant in accordance with a cutoff criterion of *P* < 0.05.

### Single-cell and single-nucleus RNA-Seq.

For single-cell RNA-Seq, cells were isolated from the mitral valve by digestion in DMEM with 1.5 mg/mL collagenase A and 0.5 mg/mL elastase for 60 minutes at 37°C. The digested tissue was passed through a 70 μm filter and incubated with cell viability dye (65-0863-14; Thermo Fisher Scientific) for 20 minutes at 4°C. Viable cells were sorted with a FACSAria (BD Biosciences) and collected in 0.4% BSA/PBS. For single-nucleus RNA-Seq, nuclei were isolated from the mitral valve using the Chromium Nuclei Isolation Kit (10x Genomics; PN-1000493). Briefly, the tissue was incubated in lysis buffer for 10 minutes at 4°C, followed by washing with debris removal and resuspension buffers. Nuclei were stained with 7AAD (Thermo Fisher) for 20 minutes at 4°C, sorted with a FACSAria, and collected in 0.4% BSA/PBS. Both single-cell and single-nucleus suspensions were used to generate RNA-Seq libraries using the Chromium Single Cell Platform (10x Genomics) according to the manufacturer’s protocol. Cells or nuclei were encapsulated into Gel Beads-in-emulsion using the Chromium system, followed by cell lysis, barcoded reverse transcription, cDNA amplification, 5′ adapter ligation, and sample index attachment. Libraries were sequenced on a HiSeq 4000 System (Illumina) at the Yale Center for Genome Analysis. Raw data were processed through a Cell Ranger (10x Genomics), and this output was further processed in R using Seurat (v4.3.0) (52). The data were filtered as follows: (a) cells expressing <200 or >5,000 genes were excluded, (b) cells with more than 10% mitochondrial gene expression were excluded, and (c) genes expressed in less than 3 cells were excluded. Gene expression was normalized by the “LogNormalize” method. Principal component analysis was conducted based on highly variable genes for dimensionality reduction, and 50 significant principal components were chosen for batch effect correction using Harmony (v0.1.1) (53). Clustering was performed using a graph-based clustering approach with appropriate resolution for each dataset. Uniform Manifold Approximation and Projection was applied for the 2-dimensional visualization of cell clustering. Genes with log fold change >0.25 and adjusted *P* < 0.05 were considered significant DEGs.

### Receptor-ligand analysis.

To identify and visualize receptor-ligand analysis among cell types, the R package CellChat (v1.5.0) was applied. The gene expression matrix was extracted from R Data Serialization (RDS) files and labeled with cell type information, and CellChat was used for deducing receptor-ligand interactions (54).

### Fibroblast cell culture and RT-PCR.

Dermal fibroblasts were obtained from ear biopsies following euthanasia from WT and mgR mice. Specimen were washed 3 times in cold PBS, transferred to cell culture dishes, covered with DMEM (Gibco; 11965-092) supplemented with 10% FBS (Gibco; 26140079) and penicillin-streptomycin (Gibco; 15140122), and cultured in an incubator at 37°C and 5% CO_2_. Fibroblasts were digested, cultured, and harvested following reaching confluence after second passage. RNA was isolated using an Qiagen RNA kit. Probe sequences for Fbn1 are as follows: forward, GCTGTGAATGCGACATGGGCTT, and reverse, TCTCACACTCGCAACGGAAGAG.

### Human specimens.

Myxomatous mitral valve tissue was obtained from patients undergoing mitral valve repair in which part of the posterior leaflet (P2) was resected or undergoing mitral valve replacement in which the posterior leaflet was excised. None of the patients had evidence of MFS or other connective tissue disorder evident by clinical phenotype or genetic testing. Normal posterior mitral valve specimens were obtained from organ donors. The specimens were processed by the investigators within the operating room to ensure precise anatomical identification.

### Statistics.

Quantitative data are presented as dot plots of individual values with bars representing the mean and SEM. Single numerical values are represented by columns. Comparison of continuous variables between 2 groups was determined by 2-tailed Student’s *t* test and for more than 2 groups by 1- or 2-way ANOVA for independent variables, followed by Tukey’s multiple-comparison tests if the null hypothesis was rejected by ANOVA. Comparison of categorical variables was by Fisher’s exact test followed by Bonferroni correction for multiple testing. Probability values were 2 tailed, and *P* < 0.05 was considered to indicate statistical significance. Graph construction and statistical analyses were performed with Prism 9.5.1 (GraphPad Software).

### Study approval.

Animal research protocols were approved by the IACUC of Yale University. Human subject research protocols with a waiver of consent were approved by the Institutional Review Board of Yale University and the New England Organ Bank.

### Data and code availability.

Values underlying the data provided in this work are available in the Supporting Data Values file. Sequencing data have been deposited at the NCBI Gene Expression Omnibus (GSE293926, GSE297923, GSE296735), and analysis codes are available at https://github.com/GaF123/ITGB1-and-mTOR-signals-in-Myxomatous-Mitral-Valve-Degeneration (commit ID: 5bfd97c, branch: main).

## Author contributions

GT, MK, YL, and AG designed the study. FG, QC, MM, and SL conducted experiments and acquired data. QC and YL analyzed RNA-Seq data. GF, MK, RF, GT, YL, and AG analyzed and interpreted data, wrote and edited the manuscript.

## Supplementary Material

Supplemental data

Supporting data values

## Version 1 05/20/2025

In-Press Preview

## Version 2 07/15/2025

Electronic publication
