# Supplementary material for: Integrin-mediated mTOR signaling drives TGF-β overactivity and myxomatous mitral valve degeneration in hypomorphic fibrillin-1 mice
Source: J Clin Invest. 2025 May 20;135(14):e183558. doi: 10.1172/JCI183558 (PMC12259269; doi:10.1172/JCI183558)
Supplement: Supplemental data [file jci-135-183558-s194.pdf]

Supplemental Materials

Integrin-Mediated mTOR Signaling Drives TGF-β Overactivity and Myxomatous Mitral Valve Degeneration in Hypomorphic Fibrillin-1 Mice

Fu Gao[1,2 3], Qixin Chen[1], Makoto Mori[1,4], Sufang Li[1], Giovanni Ferrari[4,5], Markus Krane [1,6,7], Rong Fan[2,3,8,9,10,11], George Tellides[1,12,13], Yang Liu[3,14]*, Arnar Geirsson[1,4,5,12]*

1. Division of Cardiac Surgery, Department of Surgery, Yale School of Medicine, New Haven, CT, USA.
2. Department of Biomedical Engineering, Yale University, New Haven, CT, USA.
3. Department of Pathology, Yale School of Medicine, New Haven, CT, USA.
4. Department of Surgery, Columbia University, New York, NY, USA.
5. Columbia Surgical Cardiovascular Research Institute, Columbia University, New York, USA.
6. Department of Cardiovascular Surgery, Institute Insure, German Heart Center Munich, School of Medicine & Health, Technical University of Munich, Munich, Germany.
7. DZHK (German Center for Cardiovascular Research) – partner site Munich Heart Alliance, Munich, Germany
8. Yale Stem Cell Center, Yale School of Medicine, New Haven, CT, USA.
9. Yale Cancer Center, Yale School of Medicine, New Haven, CT, USA.
10. Yale Center for Research on Aging (Y-Age), Yale School of Medicine, New Haven, CT, USA.

11. Human and Translational Immunology Program, Yale School of Medicine, New Haven, CT, USA.
12. Program in Vascular Biology and Therapeutics, Yale School of Medicine, New Haven, CT, USA
13. Veterans Affairs Connecticut Health Care System, West Haven, CT, USA.
14. Department of Neurology, Yale School of Medicine, New Haven, CT, USA.

***Corresponding authors:**

Yang Liu, PhD

Department of Pathology

Yale School of Medicine

300 George Street, New Haven, CT 06510, USA

Email: yang.liu.yl2224@yale.edu

Arnar Geirsson, MD

Division of Cardiac, Thoracic, and Vascular Surgery, Department of Surgery

Columbia University Vagelos College of Physicians and Surgeons

177 Ft Washington Ave, New York, NY 10032, USA

Telephone: +1-212-305-8312

Email: arnar.geirsson@columbia.edu

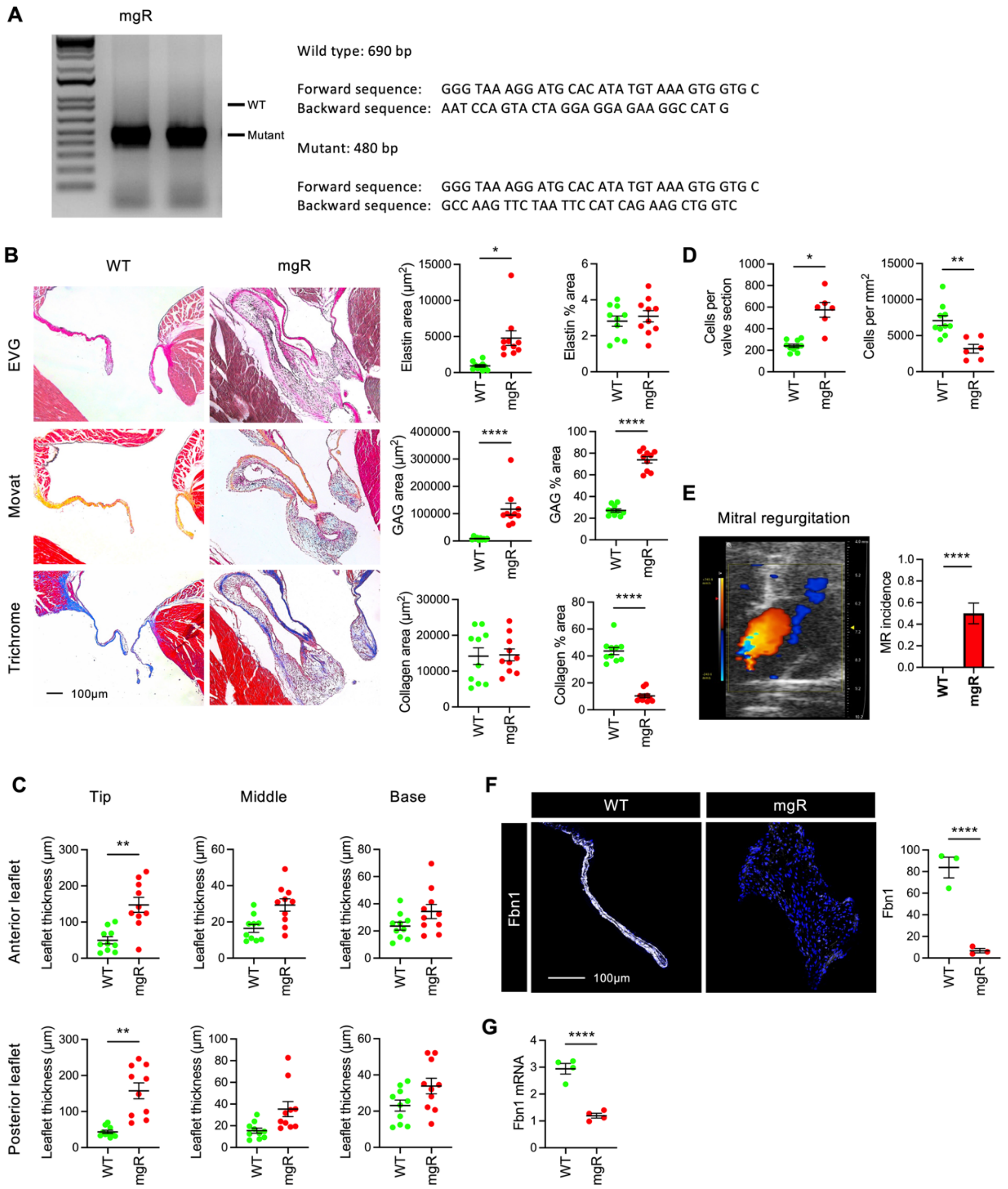

Supplemental Figure 1. Genotyping and morphometric assessment of mitral valve leaflets in WT and mgR mice at 12 weeks of age. (**A**) Genotyping PCR with probe sequences for mgR. (**B**) Elastica van Gieson (EVG), Movat pentachrome, and trichome staining to evaluate absolute and relative contents of elastin, GAGs, and collagen within mitral valve leaflets from 12-week-old WT and mgR mice, *n* = 6-10. (**C**) Comparison of maximal leaflet thickness at different locations (tip, mid, and base) of anterior and posterior mitral valve leaflets, *n* = 10. (**D**) Number of cells within valve sections and relative to total valve area, *n* = 6-10. (**E**) Representative echocardiographic image showing MR and incidence at 12 weeks of age, *n* = 26-28. (**F**) Representative IF staining and mean fluorescence density arbitrary units (a.u.) for fibrillin-1 (Fbn1), *n* = 3. (**G**) RT-PCR for Fbn1 from cultured dermal fibroblast, *n* = 4. Data are represented as individual values with mean ± SEM; *$p < 0.05$, **$p < 0.01$, ****$p < 0.0001$ by unpaired t-test.

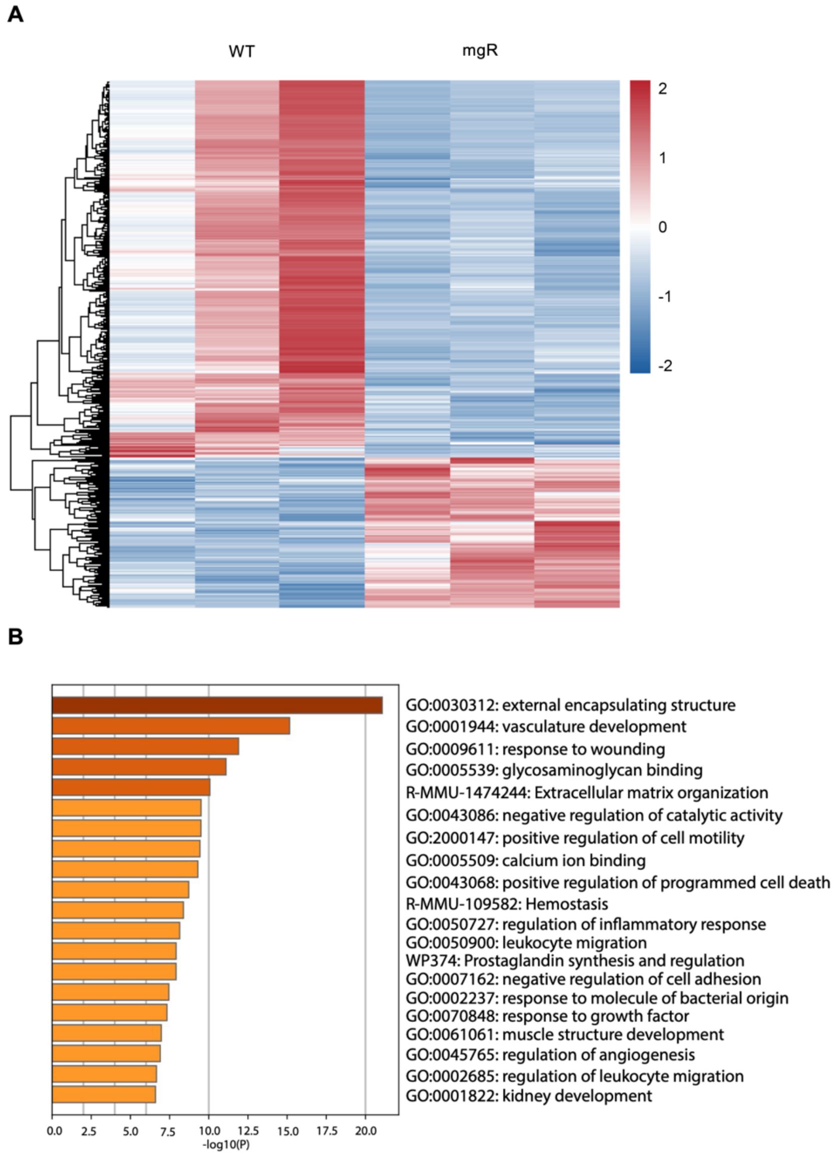

Supplemental Figure 2. Bulk RNA-Seq in WT and mgR mice at 12 weeks of age. (**A**) Heatmap with unsupervised hierarchical clustering showing differentially expressed genes (DEGs) with > 2-fold expression differences and $p < 0.05$ in mitral valves of 12-week-old WT and mgR mice; rows represent genes ($n = 1759$) and columns represent samples (n = 6), each sample pooled from 5 valve leaflets. (**B**) Gene ontology (GO) analysis showing top 20 enriched terms in mgR vs. WT mice based on DEGs with adjusted $p < 0.05$.

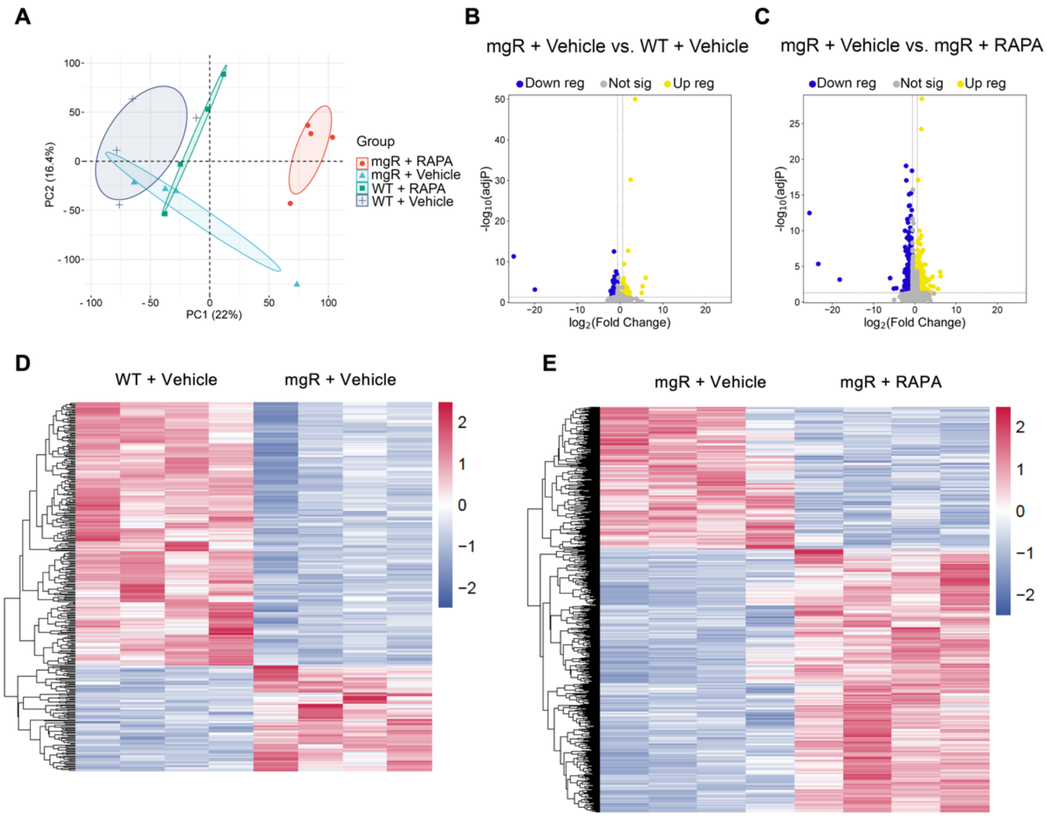

Supplemental Figure 3. Expression analysis in 5-week-old WT and mgR mice. Bulk RNA-Seq (**A**) principal component analysis of WT and mgR mice treated with vehicle or rapamycin (RAPA) for 1 week from 4 to 5 weeks of age. Volcano plots of DEGs pairwise comparison of (**B**) vehicle-treated WT vs. mgR mice and (**C**) vehicle- vs. rapamycin-treated mgR mice. Heatmap with hierarchical clustering of DEGs (> 1.5-fold expression, $p < 0.05$) in (**D**) vehicle-treated WT vs. mgR mice and (**E**) vehicle- vs. rapamycin-treated mgR mice. Rows represent genes; columns represent samples. Five valve leaflets pooled per sample, $n = 4$ samples per group.

A

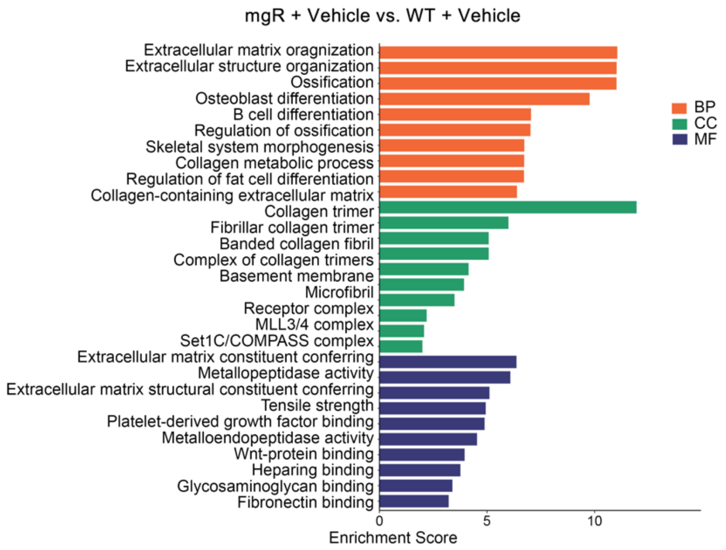

B

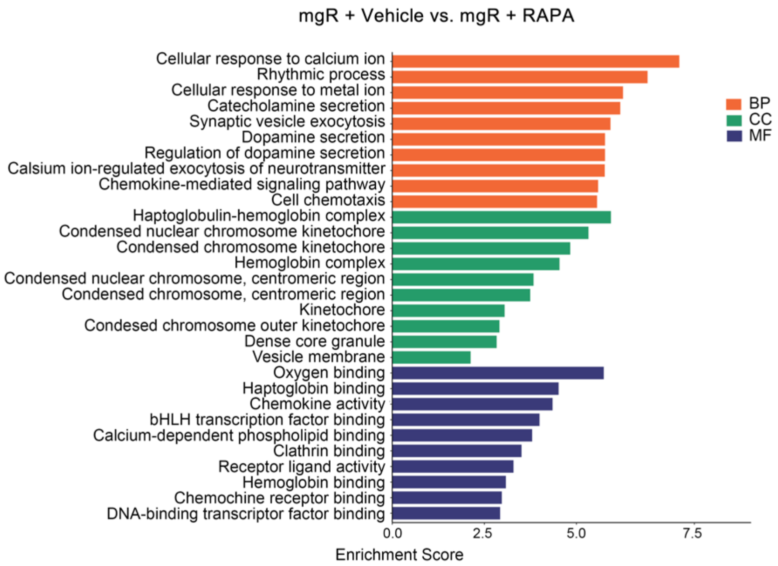

Supplemental Figure 4. Gene Ontology (GO) analysis in 5-week-old WT and mgR mice treated with vehicle or rapamycin. GO enrichment analysis of bulk RNA-Seq data for biological process (BP), cellular components (CC), and molecular function (MF) in (**A**) vehicle-treated WT vs. mgR mice and (**B**) mgR mice treated with vehicle vs. rapamycin (RAPA) for 1 week from 4 to 5 weeks of age; input genes with adjusted *p*-value < 0.05. Five valve leaflets pooled per sample, *n* = 4 samples per group.

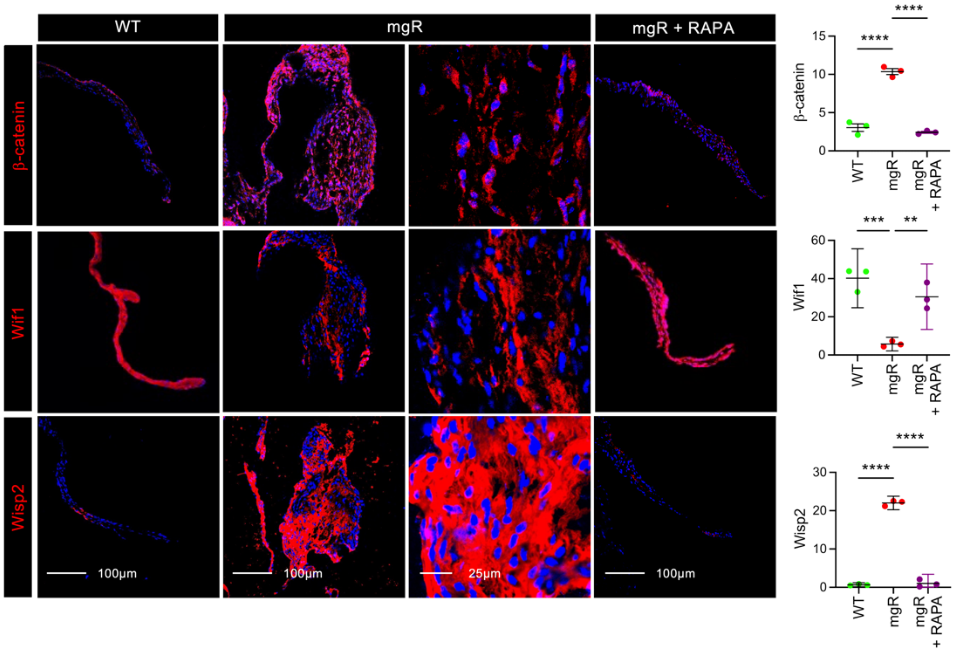

Supplemental Figure 5. Long-term treatment with rapamycin suppresses Wnt/β-catenin signaling in 12-week-old mgR mice. Representative IF staining with mean fluorescence density (a.u.) for β-catenin, Wif1, and Wisp2 in WT mice and untreated or rapamycin (RAPA)-treated mgR mice at 12 weeks of age, $n = 3$. Data are represented as individual values with mean ± SEM. $^{**}p < 0.01$, $^{***}p < 0.001$, $^{****}p < 0.0001$ by 1-way ANOVA.

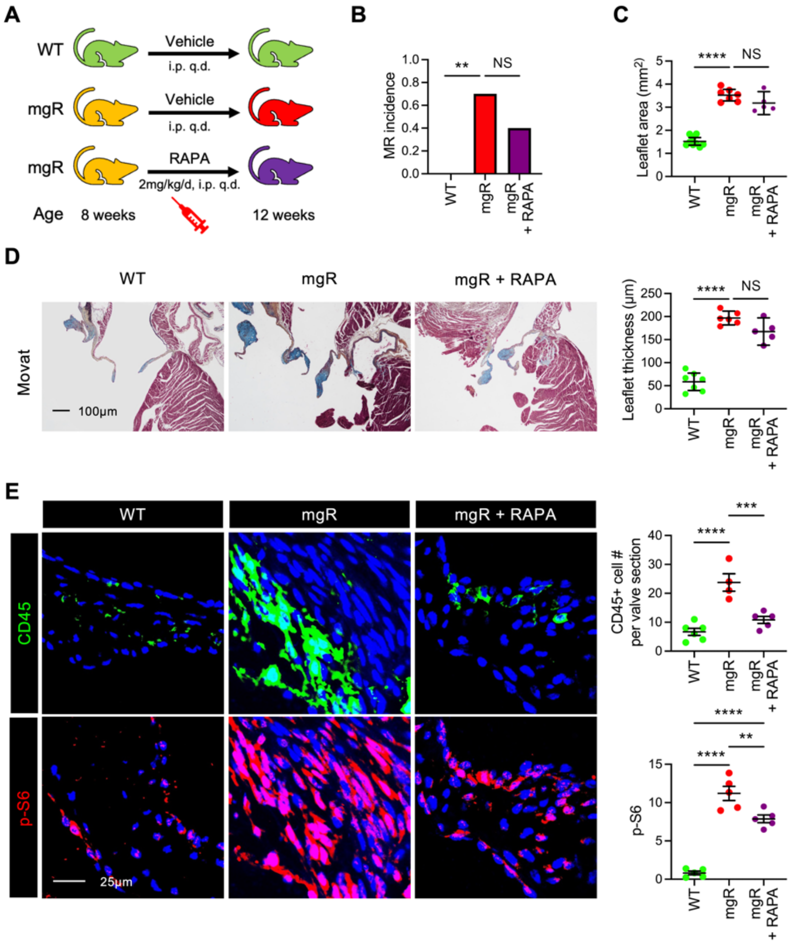

Supplemental Figure 6. Intermediate-term treatment with rapamycin in 12-week-old mgR mice. (**A**) Schema of experimental groups: WT mice were treated with vehicle and mgR mice were treated with vehicle or rapamycin (RAPA) for 4 weeks starting at 8 weeks of age. (**B**) Incidence of mitral regurgitation, *n* = 5-10. (**C**) Morphometric analysis of the anterior mitral valve leaflet area, *n* = 5-9. (**D**) Representative Movat pentachrome staining and measurement of maximal leaflet thickness, *n* = 5-7. (**E**) Representative IF staining with mean fluorescence density (a.u.) for CD45 and p-S6, *n* = 4-6. Data are represented as individual values with mean ± SEM; NS = non-significant, ***p* < 0.01, ****p* < 0.001, *****p* < 0.0001 by (**C**-**E**)1-way ANOVA or (**B**) Fisher's exact test.

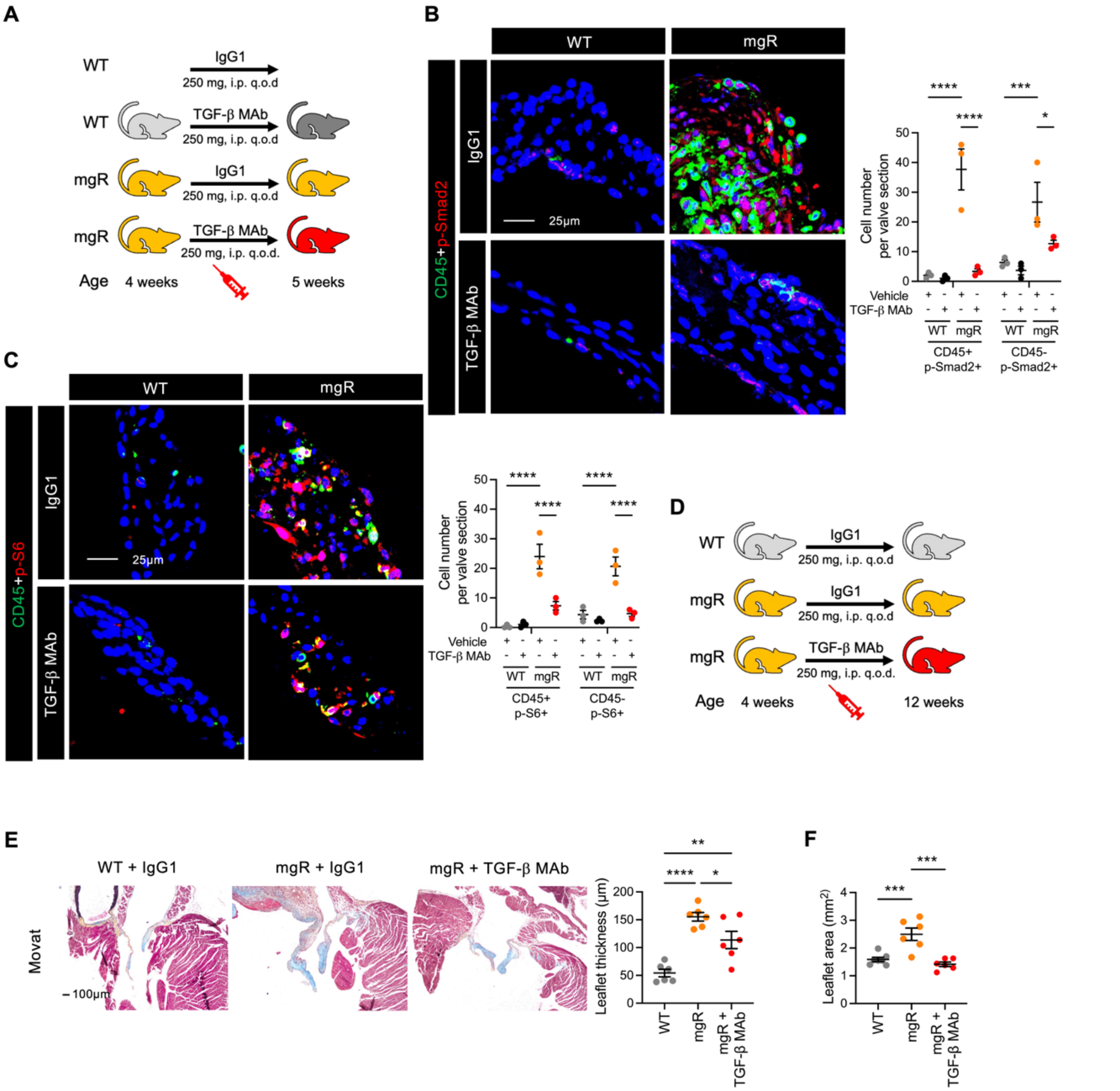

Supplemental Figure 7. TGF-β neutralization effectively inhibits SMAD2 activation resulting in partial rescue of the mitral valve phenotype in mgR mice. (**A**) Schema depicting serologic neutralization experimental design for short-term treatment in which mice were treated with either IgG1 control or TGF-β neutralizing antibody (TGF-β MAb) for 1 week starting at 4 weeks with mitral valves analyzed at 5 weeks of age. Representative IF and number of positive cells per valve section for (**B**) CD45 and p-SMAD2, and for (**C**) CD45 and p-S6 in 5-week-old mice, $n = 3$. (**D**) Schema depicting serologic neutralization experimental design for long-term treatment in which mice were treated for 8 weeks starting at 4 weeks of age with mitral valves analyzed at 12 weeks of age. (**E**) Representative Movat pentachrome staining and measurement of maximal leaflet thickness and (**F**) leaflet area in 12-week-old mice, $n = 6$-7. Data are represented as individual values with mean ± SEM; *$p < 0.05$, **$p < 0.01$, ***$p < 0.001$, ****$p < 0.0001$ by 2-way ANOVA.

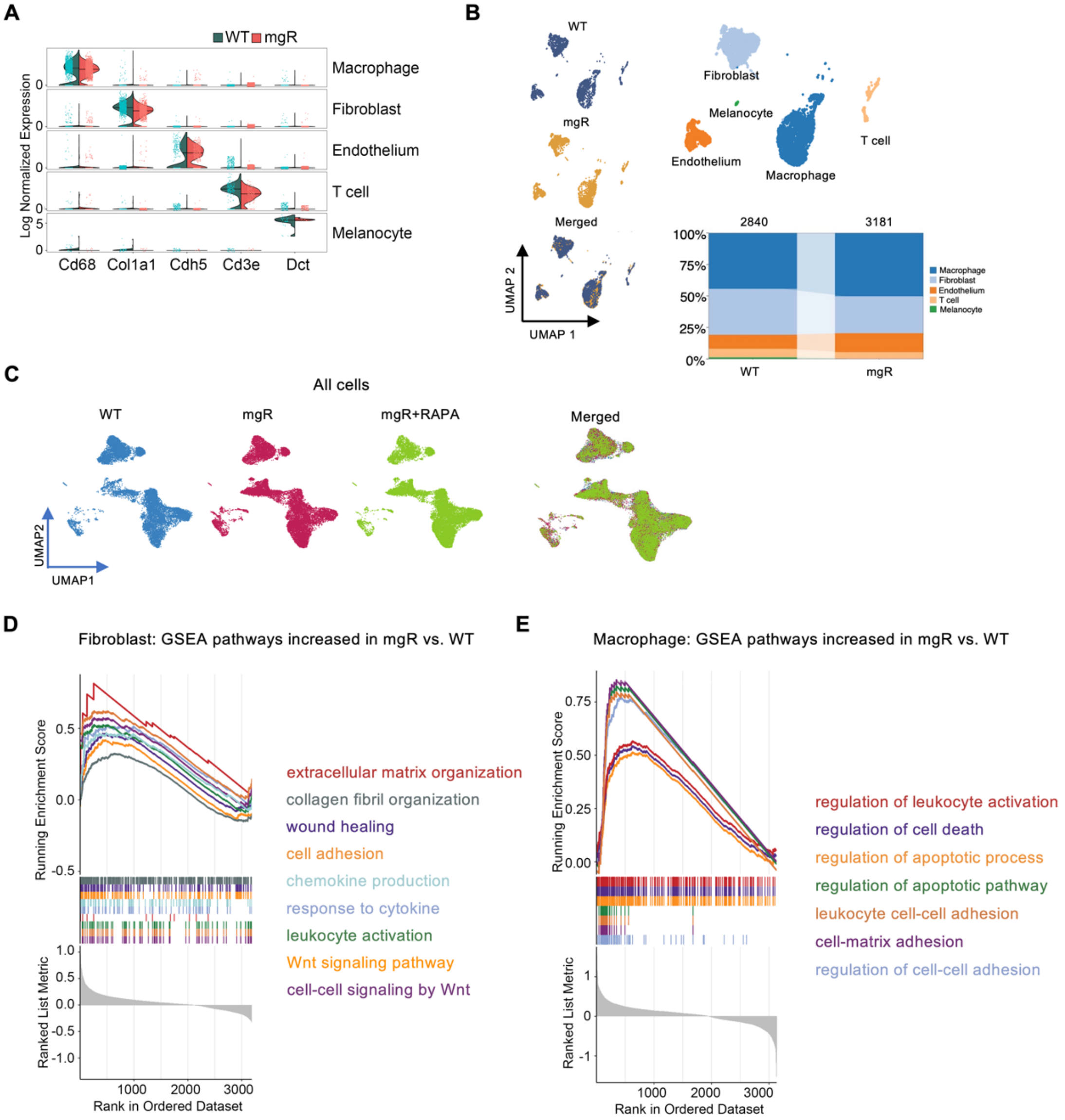

Supplemental Figure 8. Single-cell and single-nucleus RNA-Seq and pathway enrichment analysis of mitral valves from 12-week-old WT, mgR and rapamycin (RAPA) treated mgR mice. (**A**) Violin plots for characteristic marker genes across 5 cell types identified by single-cell RNA-Seq and (**B**) Uniform Manifold Approximation and Projection (UMAP) plots with annotation of primary cell types identified by single-cell RNA-Seq, demonstrating proportional differences between mitral valves from 12-week-old WT and mgR mice, *n* = 3. (**C**) UMAP plots of single-nucleus RNA-Seq from 12-week-old WT, mgR and rapamycin-treated mgR (+RAPA) mice, *n* = 20. Gene set enrichment analysis (GSEA) of single-nucleus RNA-Seq comparing 12-week-old mgR vs. WT mice for (**D**) fibroblast and (**E**) macrophage.

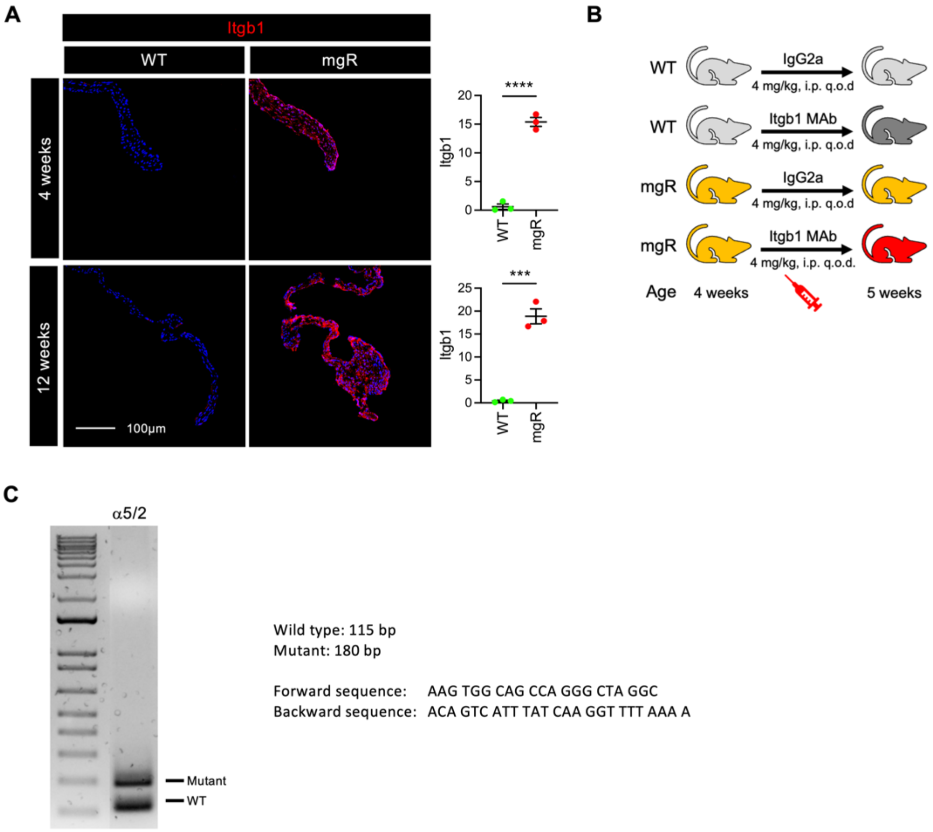

Supplemental Figure 9. Integrins in mgR mitral valve leaflets. (**A**) Representative IF staining with mean fluorescence density (a.u.) for β1-integrin (Itgb1) in 4-week-old and 12-week-old WT and mgR mice, *n* = 3. (**B**) Schema depicting serologic neutralization experimental design where mgR mice were treated with Itgb1 Mab for 1 week starting at 4 weeks with mitral valves analyzed at 5 weeks of age. (**C**) Genotyping PCR with probe sequences for α5/2. Data are represented as individual values with mean ± SEM. ***$p < 0.001$, ****$p < 0.0001$ by unpaired t-test.

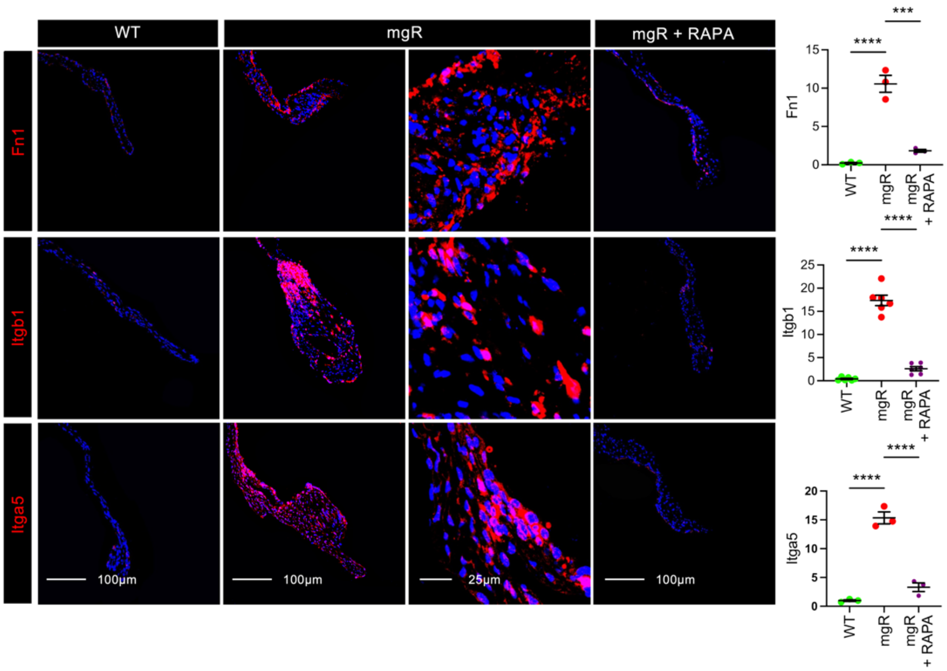

Supplemental Figure 10. Long-term treatment with rapamycin suppresses fibronectin and integrin expression in 12-week-old mgR mice. Representative IF staining with mean fluorescence density (a.u.) for fibronectin (Fn1), β1-integrin (Itgb1) and α5-integrin (Itga5) in WT mice and untreated or rapamycin (RAPA)-treated mgR mice at 12 weeks of age, n = 3-6. Data are represented as individual values with mean ± SEM. ***$p < 0.001$, ****$p < 0.0001$ by 1-way ANOVA.

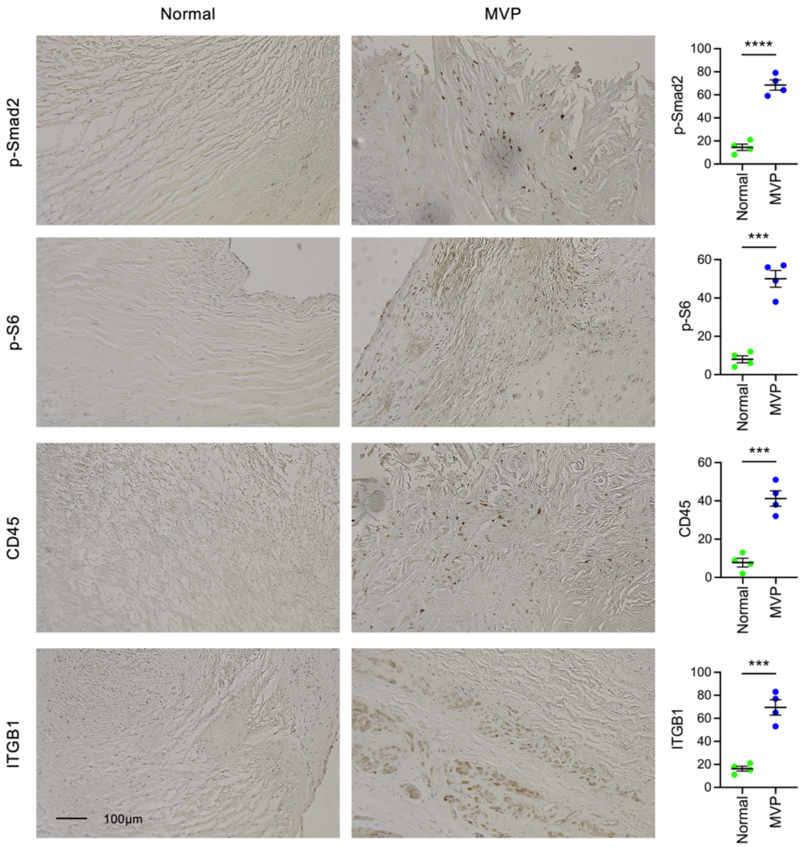

Supplemental Figure 11. Immunohistochemistry staining of human normal mitral valves and mitral valve prolapse (MVP). Representative immunohistochemistry staining with mean fluorescence density (a.u.) for p-Smad2, p-S6, CD45 and ITGB1 in human normal mitral valves and MVP, $n = 4$. Data are represented as individual values with mean ± SEM. ***$p < 0.001$, ****$p < 0.0001$ by unpaired t-test.

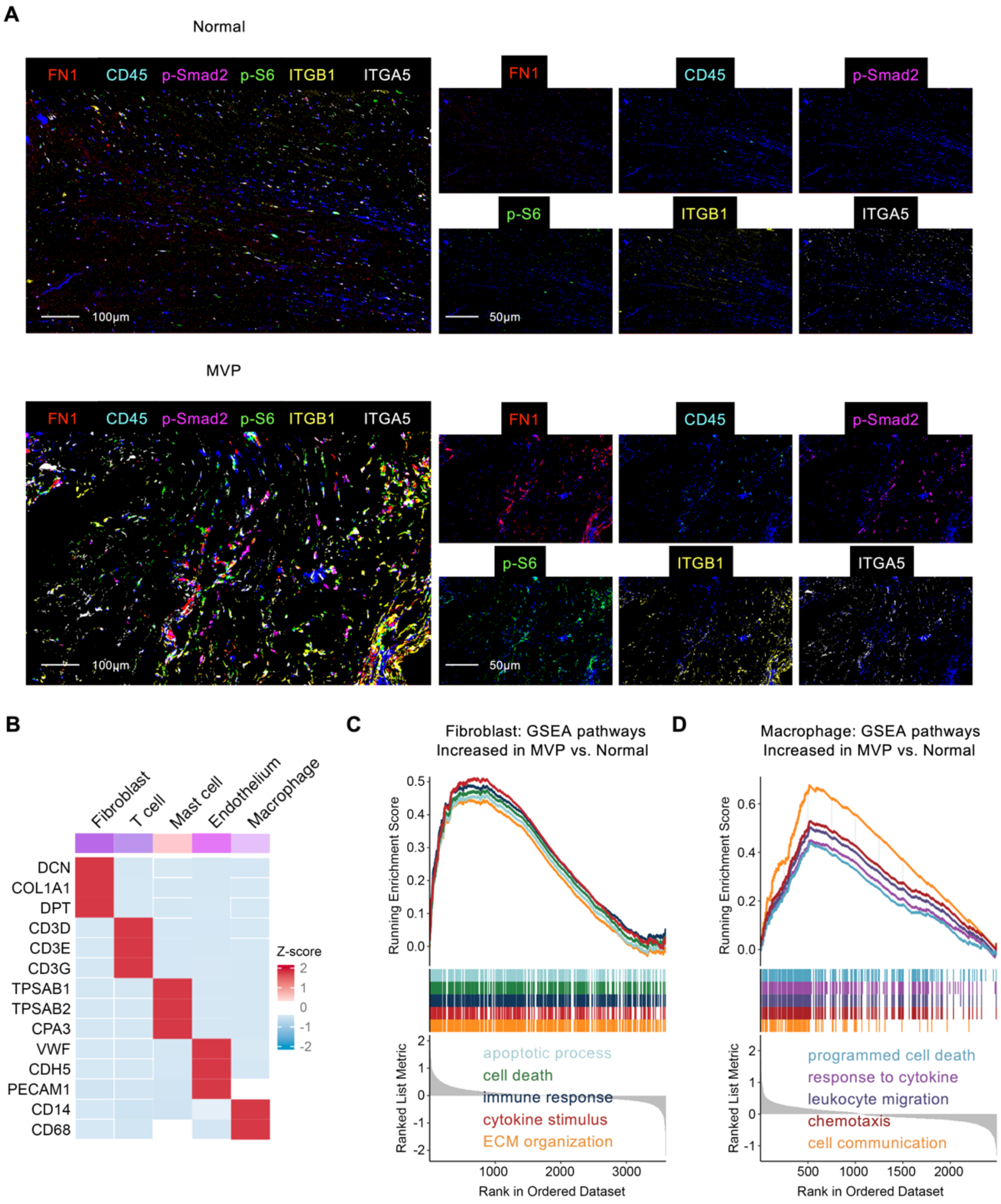

Supplemental Figure 12. Increased mTOR signaling and increased integrin expression with differential pathway enrichment in human mitral valve disease. Representative (**A**) Multiplex immunofluorescence CODEX images in human normal and MVP tissues specimen for fibronectin (FN1), CD45, p-Smad2, p-S6, β1-integrin (ITGB1), and α5-integrin (ITGA5). (**B**) Heatmap for characteristic marker genes across 5 cell types identified by single-cell RNA-Seq. Gene set enrichment analysis (GSEA) comparing MVP vs. normal in (**C**) fibroblasts and (**D**) macrophages from human mitral valve specimens, *n* = 4-6.

Supplemental Table 1. Subject demographics and medical history#

	Normal	MVP
Number	4	6
Age (yrs) (mean ± SD)	49.0 ± 5.5	58.5 ± 5.4
Male sex, No (%)	2 (50)	6 (100)
Race - white, No (%)	2 (50)	6 (100)
Active smoker, No (%)	0 (0)	0 (0)
Diabetes mellitus, No (%)	0 (0)	0 (0)
Hypertension, No (%)	0 (0)	5 (83)
Hyperlipidemia, No (%)	0 (0)	3 (50)
Chronic kidney disease, No (%)	0 (0)	0 (0)
Coronary artery disease, No (%)	0 (0)	0 (0)
Atrial fibrillation, No (%)	0 (0)	2 (33)
Aspirin, No (%)	0 (0)	1 (17)
ARB, No (%)	0 (0)	1 (17)
Beta-blocker, No (%)	0 (0)	3 (50)
CCB, No (%)	0 (0)	3 (50)

#Mitral valves were obtained from organ donors without disease or after surgical repair or replacement for myxomatous mitral valve disease. MVP: mitral valve prolapse; yrs: years; ARB: angiotensin receptor blocker; CCB: calcium channel blocker.
